# Primate Amygdala Neurons Simulate Decision Processes of Social Partners

**DOI:** 10.1016/j.cell.2019.02.042

**Published:** 2019-05-02

**Authors:** Fabian Grabenhorst, Raymundo Báez-Mendoza, Wilfried Genest, Gustavo Deco, Wolfram Schultz

**Affiliations:** 1 Department of Physiology, Development and Neuroscience, University of Cambridge, Downing Street, Cambridge CB2 3DY, UK; 2 Center for Brain and Cognition, Department of Technology and Information, Universitat Pompeu Fabra, Carrer Tànger, 122-140, 08018 Barcelona, Spain; 3 Institució Catalana de la Recerca i Estudis Avançats, Universitat Barcelona, Passeig Lluís Companys 23, 08010 Barcelona, Spain

**Keywords:** reward, decision-making, observational learning, social cognition, mirror neuron, autism, theory of mind, attractor network

## Abstract

By observing their social partners, primates learn about reward values of objects. Here, we show that monkeys’ amygdala neurons derive object values from observation and use these values to simulate a partner monkey’s decision process. While monkeys alternated making reward-based choices, amygdala neurons encoded object-specific values learned from observation. Dynamic activities converted these values to representations of the recorded monkey’s own choices. Surprisingly, the same activity patterns unfolded spontaneously before partner’s choices in separate neurons, as if these neurons simulated the partner’s decision-making. These “simulation neurons” encoded signatures of mutual-inhibitory decision computation, including value comparisons and value-to-choice conversions, resulting in accurate predictions of partner’s choices. Population decoding identified differential contributions of amygdala subnuclei. Biophysical modeling of amygdala circuits showed that simulation neurons emerge naturally from convergence between object-value neurons and self-other neurons. By simulating decision computations during observation, these neurons could allow primates to reconstruct their social partners’ mental states.

## Highlights

• Amygdala neurons derive object values from own experience and social observation
• Simulation neurons convert object values to predictions of social partner’s choices
• Simulation neurons code signatures of decision computation during social observation
• Simulation signals can emerge from converging value signals and self-other signals

## Graphical Abstract

**Figure:**
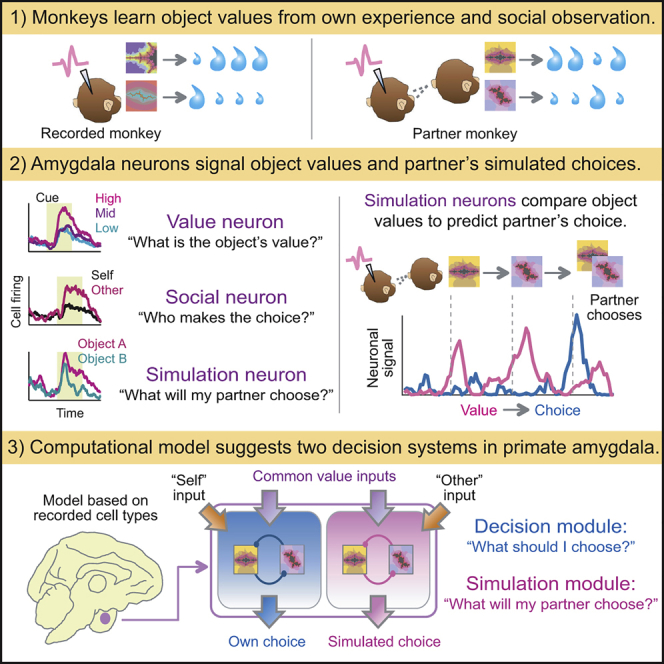

## Teaser

When monkeys observe and learn from each other’s choices, neurons in the amygdala spontaneously encode decision computations to simulate the social partner’s choices.

## Introduction

Primates observe the choices of social partners to learn about the reward value of objects. Such values learned from observation not only inform own decision-making but may also provide a basis for understanding the decisions of others. For example, by observing partners’ foraging choices, primates learn which foods are valuable and worth choosing (van de Waal et al., 2013). In turn, knowing how a partner values specific objects may help the observer to model the partner’s future decisions (Barlow, 1990, Lee and Seo, 2016). These cognitive processes—learning from others and predicting their choices—are critical foundations for primates’ sophisticated social behavior. Yet, despite recent progress in primate social neuroscience (Adolphs, 2006, Chang et al., 2013a, Isoda et al., 2018, Lee and Seo, 2016, Wittmann et al., 2018), their neuronal basis is poorly understood.

Neurophysiological recordings in primates have shown that neurons in select brain structures encode the observed actions (Báez-Mendoza et al., 2013, Fabbri-Destro and Rizzolatti, 2008, Yoshida et al., 2011), performance errors (Báez-Mendoza and Schultz, 2016, Yoshida et al., 2012), and expected rewards (Chang et al., 2013b, Chang et al., 2015) of social others. One recent study identified neurons that explicitly predicted others’ choices in a strategic game (Haroush and Williams, 2015). While these signals constitute important building blocks for social behavior, several key questions remain open.

First, the neuronal value inputs leading to social choice predictions are unclear. Reinforcement learning provides a mechanism whereby neurons can derive values for decision-making from past choices and experienced outcomes (Schultz et al., 1997, Sutton and Barto, 1998). Such values may also be learned from observing social partners, likely through the same associative processes (Behrens et al., 2008, Heyes, 2012). However, it is unknown whether neurons indeed derive object values from social, observational learning, and whether a shared code underlies both observation-derived and experience-derived values.

Second, the neuronal mechanisms that translate values to social choice predictions are unknown. Cognitive theories suggest that understanding others’ decisions requires simulation by the same mechanisms underlying one’s own mental states (Adolphs, 2006, Gordon, 1996, Shanton and Goldman, 2010). In neural networks, decision-making involves mutual-inhibitory competition between choice-coding neurons, which signal this competition as dynamic value comparisons and value-to-choice conversions (Deco et al., 2013, Hunt and Hayden, 2017, Tsutsui et al., 2016, Wang, 2008). But whether these neuronal computations also underlie modeling of social partners’ decisions, as implied by the simulation view, has never been tested.

We reasoned that the amygdala—a collection of nuclei in the temporal lobe—may be important in these processes. Amygdala neurons process associatively learned values (Chang et al., 2015, Johansen et al., 2011, Paton et al., 2006), economic decisions (Grabenhorst et al., 2012, Grabenhorst et al., 2016), and social information, including faces (Gothard et al., 2007, Leonard et al., 1985, Munuera et al., 2018, Rutishauser et al., 2013). Amygdala damage profoundly impairs primates’ social behavior (Adolphs et al., 1998, Adolphs et al., 2005, Kluver and Bucy, 1939). The amygdala is also implicated in autism (Amaral et al., 2008, Baron-Cohen et al., 2000, Rutishauser et al., 2013), which is marked by impoverished social cognition (Lai et al., 2014). Although the amygdala’s role in social behavior is typically explained in terms of associative learning and social perception, whether amygdala neurons also contribute to more complex social cognition is unclear.

To address these questions, we recorded the activity of single amygdala neurons in a social context in which two monkeys observed and learned from each other’s reward-based choices. We found that amygdala neurons encoded object-specific values learned from social observation and own experience in a common code, as suitable decision inputs. Distinct “simulation neurons” dynamically translated these values into representations of the partner monkey’s forthcoming choices. Beyond choice predictions, these neurons encoded the critical well-conceptualized signatures of a neuronal decision computation, specifically before the partner’s (but not recorded monkey’s) decisions. Based on these single-neuron data, we propose a biophysically realistic theory of mental simulation as neural decision computation. We show that simulation neurons emerge naturally via self-organization from object-value neurons and additionally found self-other discriminating neurons.

Our data and model suggest that distinct neurons in primate amygdala use common value inputs to compute own decisions and simulate decisions of social partners. By encoding decision computations during social observation, amygdala simulation neurons could constitute basic precursors for human mentalizing capacities.

## Results

### Observational Learning Task and Behavior

**Figure 1:**
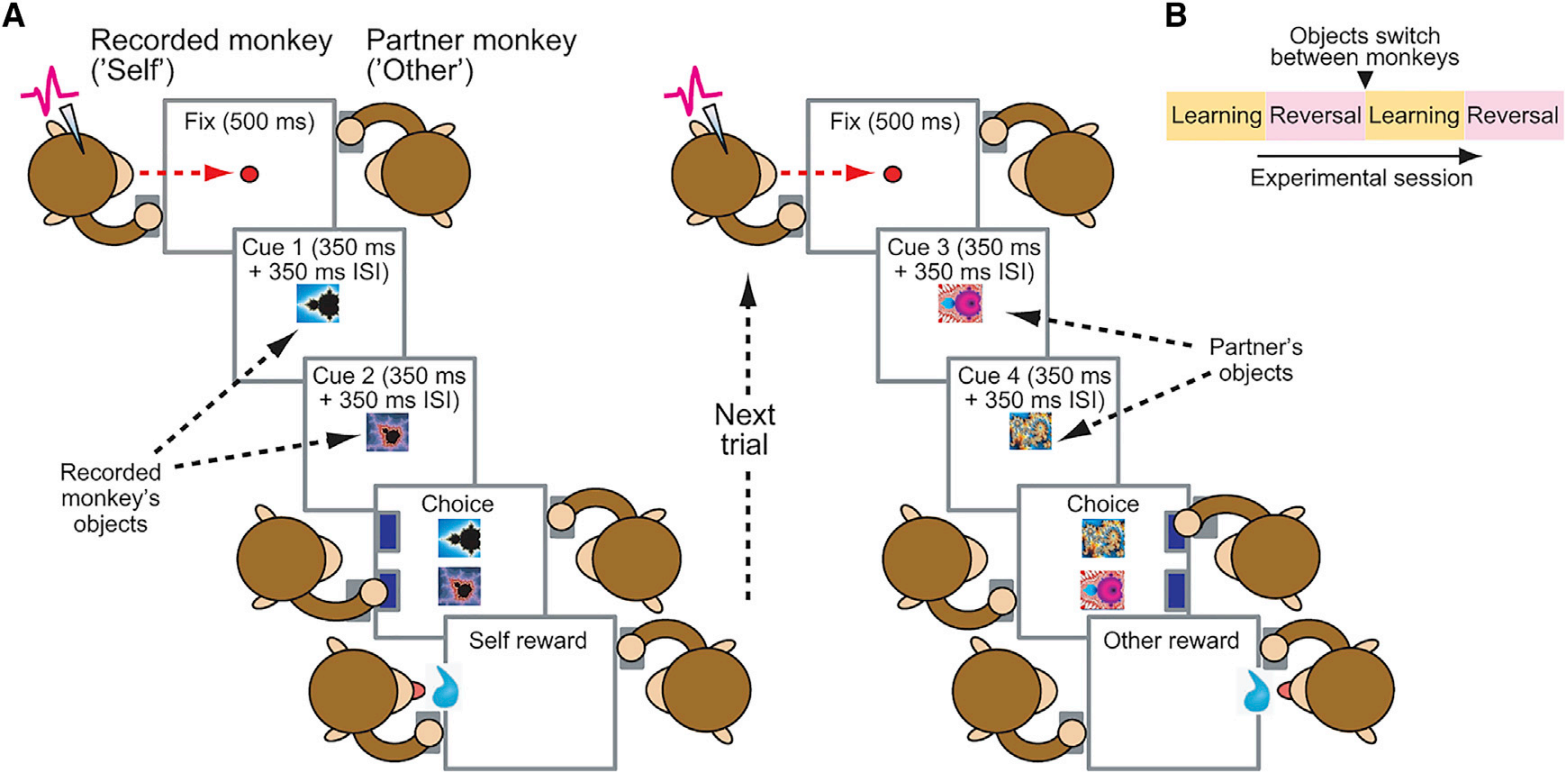
Observational Learning Task (A) Task. Two monkeys faced each other over a touch screen and took turns making choices between sequentially shown visual objects to learn object-reward probabilities (object values). The recorded monkey was required to fixate the screen center on own (self) and partner’s (other) trials until blue touch targets appeared. ISI, inter-stimulus interval. (B) Design for a testing session. Initial learning of object-reward probabilities was followed by reward-probability reversal (testing value-tracking) and object switch between animals (testing observation learning).

We studied the neuronal basis of observational learning in single amygdala neurons while two monkeys observed and learned from each other’s choices for different visual cues (“objects”) (Figure 1A). The task allowed the animals to track the changing reward probabilities of choice objects (“object values”) for themselves and their partner. Importantly, the two animals worked on distinct object sets. To encourage observational learning, we switched object sets between animals (Figure 1B) and tested whether prior observation of partner’s choices benefitted own performance. We also reversed object-reward probabilities to test value tracking. (We use the term “switch” to refer to object switches between animals.)

**Figure 2:**
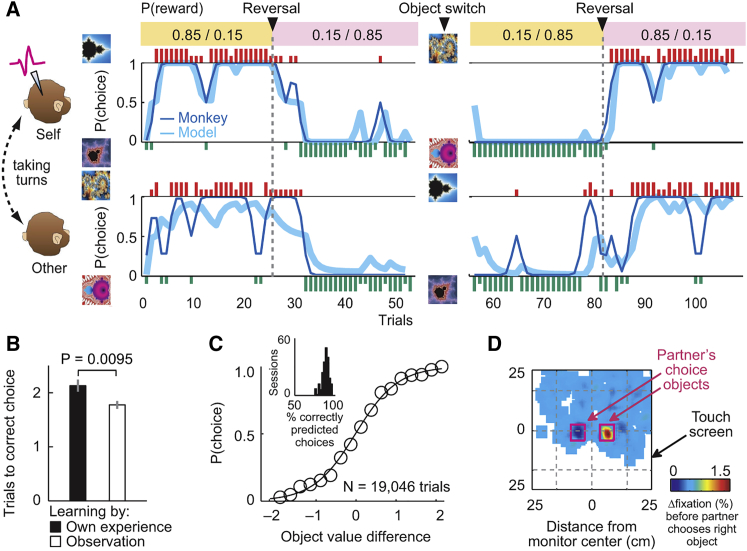
Monkeys Observe and Learn from Each Other’s Choices (A) Example session. Trial-by-trial record of choices and rewards for recorded monkey (top panels) and partner (bottom panels). Blue curves, seven-trial running averages of choices (dark) and modeled choice probabilities (light); vertical bars, single-trial choices, referenced to objects on left of each panel; short and long bars, unrewarded and rewarded choices. Numbers in colored boxes indicate object-reward probabilities. (B) Observational learning in choices. Number of trials required for first choice of high-probability object, comparing initial trials (learning from experience) and post-switch trials (after observing partner’s choices, t(186) = 2.62, paired t test). (C) Reinforcement learning model. Psychometric function relating model-derived value difference to choice probability (across animals and sessions, error bars smaller than symbols) is shown. Inset: histogram of correctly modeled choices. (D) Observational learning in gaze patterns. Contrast map of recorded monkey’s fixations before partner’s right versus left choices (measured after appearance of both objects before partner released touch key). The monkey was more likely to fixate on the object the partner was going to choose, before the partner’s movement (p < 1.0 × 10^−16^, rank-sum test). All averages are mean ± SEM. See also Figure S1.

**Figure S1:**
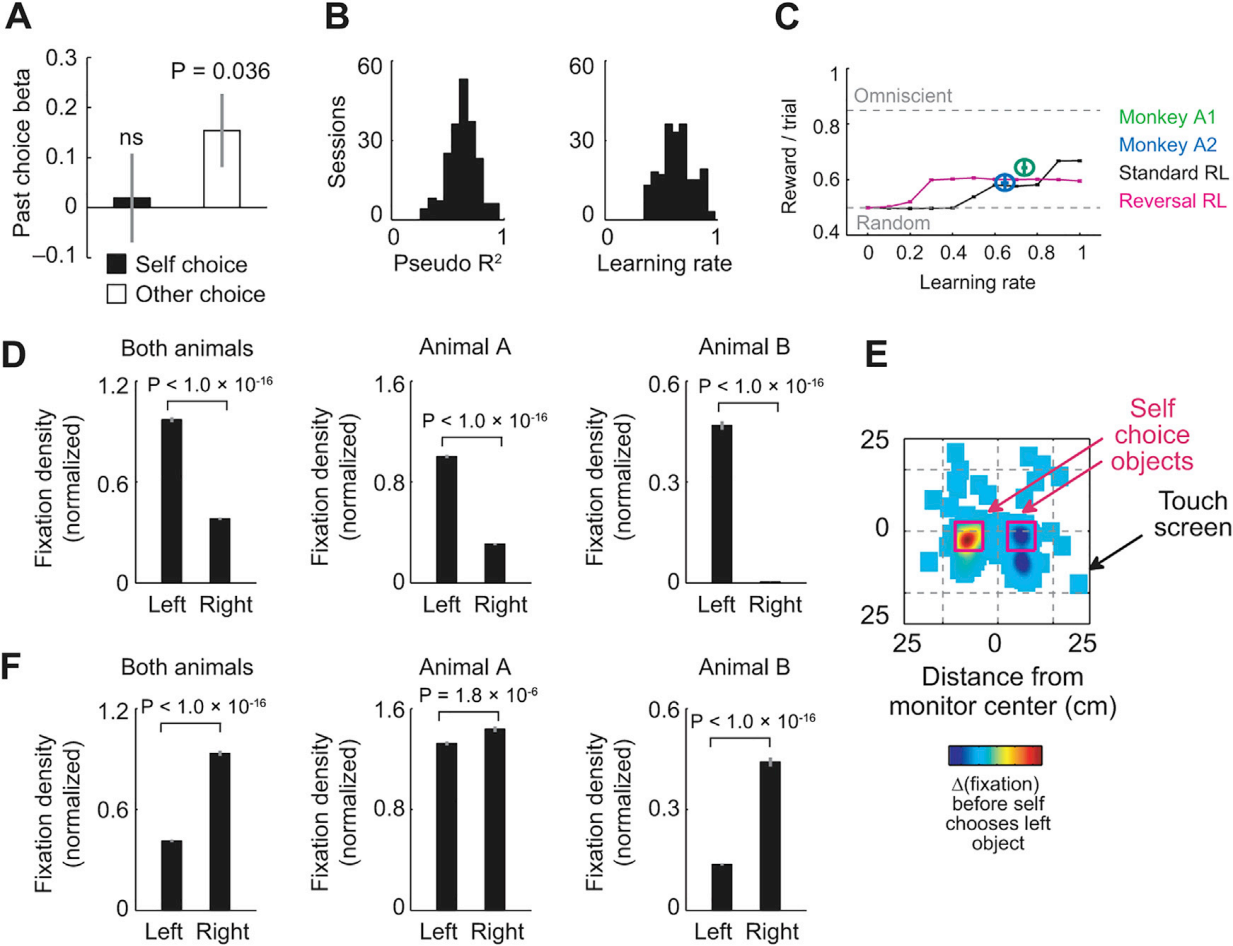
Behavioral Data, Related to Figure 2 **A,** Recorded monkey’s choices after object switch depended on partner’s immediately preceding pre-switch performance. Regression of recorded monkey’s post-switch choice probability for the high-value object (calculated over the first three post-switch trials) on partner’s pre-switch choice probability for the same object (‘Other choice’) and, as control, for the recorded monkey’s pre-switch choice probability for a different high-value object (‘Self choice’). The recorded monkey was more likely to choose the high-value object if the partner chose the same object immediately before switch. **B,** Results of fitting reinforcement learning models. Distributions of pseudo R^2^ and learning rate across animals and sessions. **C,** Monkey’s behavior matched reinforcement learners. Reward rate obtained by both recorded monkeys (green, blue) within the first three trials post-switch (mean ± s.e.m. across sessions), and best-fitting reinforcement learning model plotted for different learning rates (Equation 1). Black and magenta curves show corresponding reward rate (mean ± s.e.m) of simulated reinforcement learner within three simulated post-switch trials (10,000 iterations). ‘Random’: reward rate obtained by agent making random choices; ‘Omniscient’: reward rate obtained by agent always choosing the best option. Choice stochasticity parameter for reinforcement learner was set to the mean parameter value of the animals. **D,** Fixations for left and right object on own left-choice trials. Fixation densities for regions of interest corresponding to location of left and right object, measured after cue appearance before left-object choice, across animals (left) and separately for both recorded monkeys (fixations were not measured for non-recorded, partner monkey). Statistical test: ranksum test. **E,** Contrast map of recorded monkey’s fixations before recorded monkey’s right versus left choices (measured after target appearance before recorded monkey released touch key). **F,** Fixations for left and right object on partner’s right-choice trials (left/right refers to the recorded monkey’s perspective). Fixation densities for regions of interest corresponding to location of left and right object measured after cue appearance before partner’s choice. (As both animals faced each other over the touch screen, partner’s left choice corresponds to observed right choice for recorded monkey.) All averages are mean ± s.e.m.

In single sessions, the monkeys’ choices tracked object values over probability reversals and object switches between animals (Figure 2A). On average, the animals required fewer trials to choose the best object post-switch (i.e., after objects had switched between animals) compared to individual learning (Figure 2B), depending on the partner’s preceding performance (Figure S1A). The animals’ choices were well described by reinforcement learning models that estimated subjective values from each animal’s own choice-reward history (Figures 2C and S1B; Table S1; Equations 1 and 2). The animals’ performance approximated optimality by matching model performance (Figure S1C). Value learning was also expressed in gaze patterns: on own trials, the animals looked longer at objects they were going to choose (Figures S1D and S1E); on partner’s trials, they looked longer at objects the partner was going to choose, thereby anticipating the partner’s choices (p < 1.0 × 10^−16^; Figures 2D and S1F). Thus, monkeys learned object values from observation and used these values for own decision-making and for predicting their partner’s choices.

### Amygdala Neurons Signal Object Values Learned from Observation and Own Experience in a Common Code

**Figure 3:**
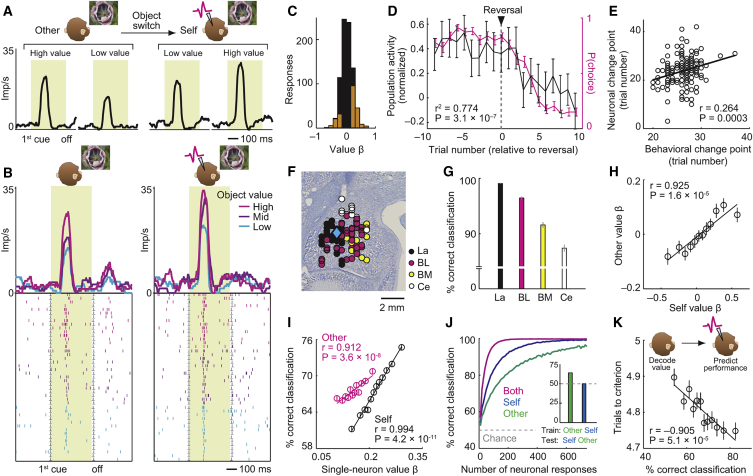
Amygdala Neurons Encode Object Values from Observational Learning and Own Experience in a Common Code (A) A single amygdala neuron, recorded in lateral nucleus, tracked object-reward probability on partner’s trials and recorded monkey’s trials. (B) Subjective value coding. The neuron in (A) encoded trial-by-trial subjective values derived from reinforcement models. Peri-event time histogram sorted by value terciles is shown. Raster display: ticks indicate impulses, rows indicate trials. (C) Histogram of value slopes (β) for all responses (black) and value-coding responses (orange). (D) Activity of value-coding neurons on partner’s trials (black) tracked partner’s choice probability (magenta) when reward probabilities reversed. (E) Neuronal-behavioral correspondence. Value-coding neurons’ change points tracked behavioral change points. (F) Reconstructed locations of value-coding neurons, superimposed on cresyl violet-stained section through one animal’s amygdala. Colors indicate different nuclei (La, lateral; BL, basolateral; BM, basomedial; Ce, centromedial). Diamond: neuron from (A) and (B). Collapsing in anterior-posterior dimension resulted in symbol overlap. (G) Value decoding across nuclei. Leave-one-out cross-validated accuracy of support-vector-machine classifier decoding high versus low value from 20 highest-slope neurons per nucleus, using data from both animals (all differences: p < 0.005, Wilcoxon test). (H) Single-neuron value slopes for recorded monkey and partner (linear regression). (I) Classifier value decoding depended on single-neuron value slopes for self and other. Decoding is based on randomly sampled subsets of 20 neurons (5,000 iterations). (J) Value decoding on recorded monkey’s and partner’s trials. (Decoding is from 205 neurons × 4 objects.) Inset: successful decoding when training classifier on partner’s data before object switch to decode (test) recorded monkey’s post-switch values (green, values supporting own observation learning) but not vice versa (blue, values irrelevant for own observation learning). (K) Relationship between observation-derived neuronal values and observation-learning performance. Across sessions, decoding accuracy for partner’s values before object-switch predicted recorded monkey’s post-switch learning. Subset-decoding as in (I). All averages are mean ± SEM. p values indicate Pearson correlation. See also Figure S2.

Individual amygdala neurons signaled reward values for specific objects, irrespective of whether these values derived from own experience or from observed partner’s choices. On partner’s trials, the neuron in Figure 3A responded strongly when the object’s value was high and the partner chose it frequently. Responses declined after unannounced probability reversal when the partner eventually preferred the alternative. After objects switched between animals, the neuron continued to track value during the recorded monkey’s own choices. These neuronal responses reflected the object’s subjective value derived from reinforcement models fitted separately to partner’s and recorded monkey’s reward-choice histories (Figure 3B; p = 0.0001, multiple regression, Equation 3).

**Figure S2:**
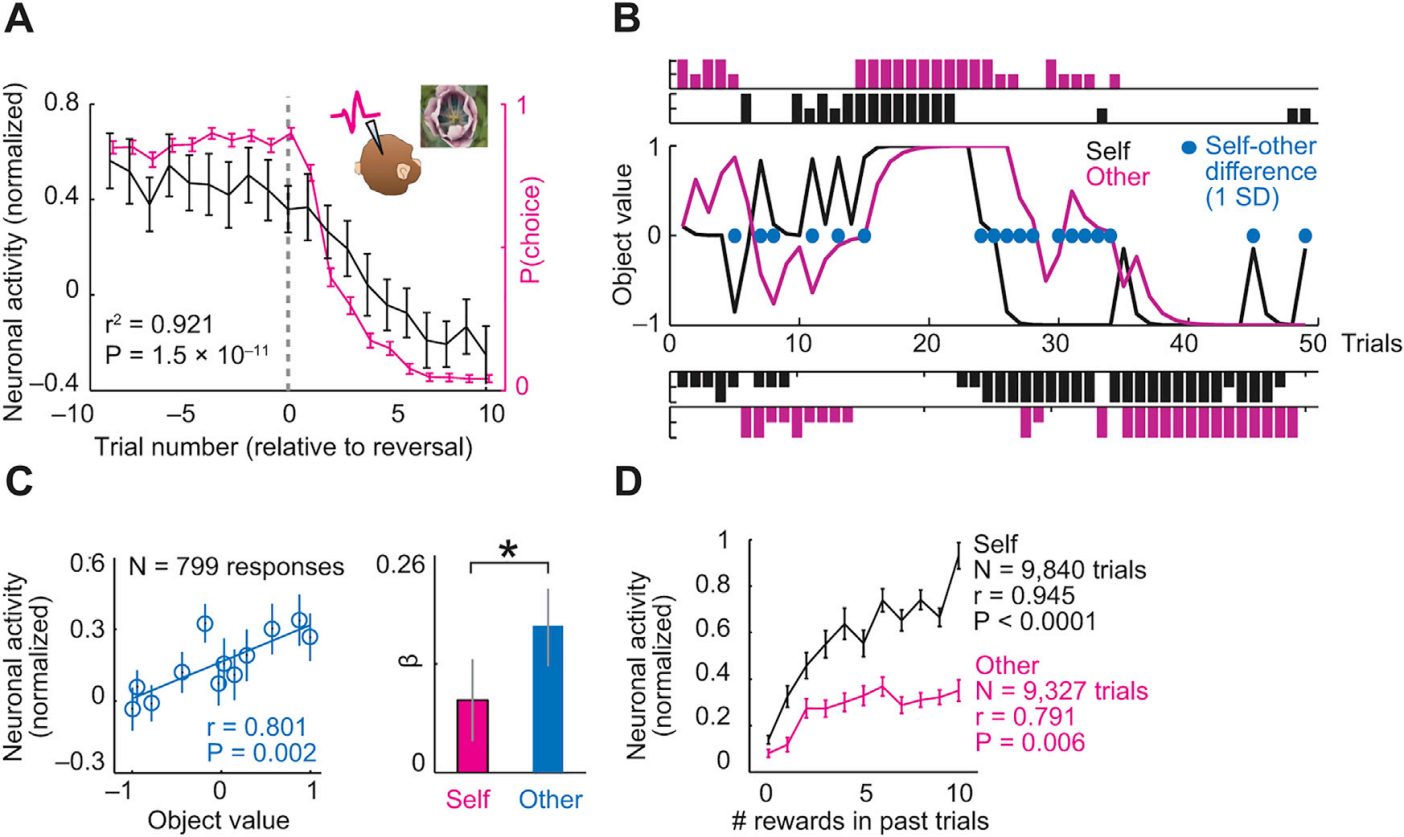
Object-Value Coding Tests, Related to Figure 3 **A,** Activity of value-coding neurons on recorded monkey’s trials (black) tracked recorded monkey’s choice probability (magenta) over reward-probability reversal. **B, C,** Control analysis for encoding of partner’s values versus recorded monkey’s values. **B,** Trial-by-trial record of subjective values, choices and rewards in an example session for recorded monkey (black) and partner (magenta). Curves: subjective object values derived from reinforcement learning model fitted to data on recorded monkey’s trials (black) and partner’s trials (magenta). Blue points indicate trials with significant (larger than one standard deviation) value difference between animals; these values (across recording sessions) and corresponding neuronal activities were used for analysis in (C). Vertical bars: single-trial choices, referenced to specific objects (upper versus lower panels); short/long bars: unrewarded/rewarded choices. **C,** Better relationship between neuronal data on partner’s trials and partner’s object values, compared to recorded monkey’s object values. Left: linear regression of neuronal responses on partner’s trials on partner’s object values, calculated across sessions and neurons, for trials with significant difference between partner’s and recorded monkey’s values (blue data points in (B)). Right: Comparison between regression coefficients calculated for partner and recorded monkey (p < 0.005). The relationship between neuronal activity on partner’s trials and partner’s object values was not explained by relationship to recorded monkey’s own values. Thus, when subjective values differed markedly between animals (due to different choice-reward histories), responses on partner’s trials distinctly reflected partner’s values rather than recorded monkey’s values. **D,** Population activity separately reflected reward-choice histories of partner and recorded monkey. Neuronal object responses on recorded monkey’s trials (black) and partner’s trials (magenta) for different numbers of rewards recently received from object choices. Object responses on both partner’s trials and recorded monkey’s trials were stronger for more frequently rewarded objects. Thus, neurons were directly sensitive to partner’s reward history separately from recorded monkey’s own reward history. All averages are mean ± s.e.m.

Among 205 recorded amygdala neurons, 127 neurons (62%) encoded such values (Figure 3C), often specifically for one object (70/127 neurons, 55%, Table S2). We identified these neurons by first selecting object-evoked responses (p < 0.005, Wilcoxon test) and then regressing these responses on model-derived subjective values, controlling for self-other trial type, object choice, and object sequence (p < 0.05, multiple regression, Equation 3). Population activity on partner’s trials closely followed the changing subjective values in step with the partner’s choices (Figures 3D and S2A). Change-point analysis (Paton et al., 2006) further confirmed this neuronal-behavioral correspondence (Figure 3E). Importantly, value signals for partner’s objects appeared even before the recorded monkey experienced reward from these objects (46/205 neurons, 22%, Equation 4); such signals thus derived purely from observation, as supported by further analyses (Figures S2B–S2D). Although value-coding neurons were present across different amygdala nuclei (Figure 3F) (Paxinos et al., 2000), support-vector-machine classification showed the strongest value signals in the lateral nucleus (Figure 3G), a key region for associative learning (Johansen et al., 2011). Thus, amygdala neurons derived object values from own experience and observational learning.

We tested whether neurons encoded observation-derived values and experience-derived values in a shared code, which would facilitate their flexible use as decision inputs. Consistent with shared encoding, single-neuron value slopes calculated separately for recorded monkey’s and partner’s trials were highly correlated (Figure 3H). Support-vector-machine classification demonstrated that value-coding precision depended on both animals’ data and that precision increased with the number of neurons in the decoding sample (Figures 3I and 3J). Training the classifier to decode partner’s values before object switch (i.e., during observational learning) allowed significant cross-decoding of the recorded monkey’s values post-switch (Figure 3J, inset). In other words, value signals on partner’s trials during observation could be read out using the recorded monkey’s own value code. These findings indicated a shared, transferable value code in amygdala.

Crucially, value-coding precision on partner’s trials predicted the recorded monkey’s own performance: on average, when the recorded monkey’s neurons showed more precise value coding during the initial observation phase (before object switch), the recorded monkey learned faster once objects had switched (Figure 3K). Thus, observation-derived neuronal values were behaviorally relevant and constituted suitable inputs for decision-making.

### Amygdala Neurons Simulate Decision Processes during Social Observation

**Figure S3:**
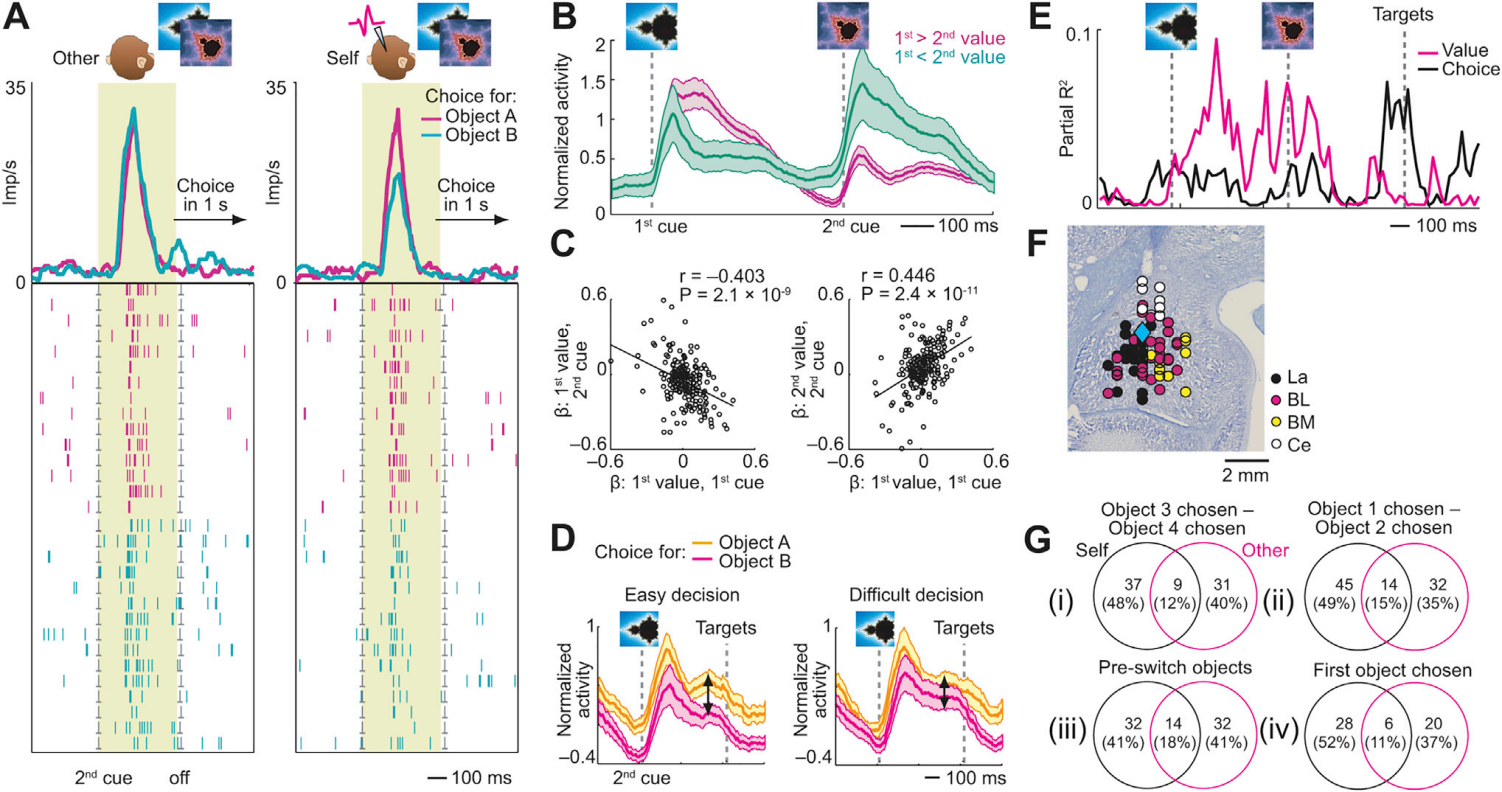
Amygdala Neurons Signal Recorded Monkey’s Decision Processes, Related to Figure 4 **A,** Single neuron predicting choice for recorded monkey but not partner. Responses to second object on partner’s trials (left) and recorded monkey’s trials (right), sorted by forthcoming object choice. **B,** Neuronal value comparison on recorded monkey’s trials. Population activity of value-coding neurons (N = 107, Equation 9) during sequential presentation of recorded monkey’s choice objects, sorted according to value of first object. **C,** Neuronal value slopes on recorded monkey’s trials indicate mutual-inhibitory value comparison. Left: anti-correlated value slopes for first object at first cue and second cue; value signals changed sign from first to second cue. Right: correlated values slopes for first and second object. **D,** Stronger choice signals for easier decisions. Population activity of choice-predictive neurons on recorded monkey’s trials for easy and difficult decisions (median-split by value difference). **E,** Single-neuron value-to-choice transition on recorded monkey’s trials. Explained variance of value and choice regressors from sliding-window regression. Activity transitioned from coding value to predicting recorded monkey’s choice, indicative of decision-making. **F,** Location of neurons that predicted recorded monkey’s choices. Diamond: neuron from (A). All averages are mean ± s.e.m. **G,** Assessing joint versus separate choice coding for self and other. Numbers of neurons encoding choices for self only (black circles), other only (magenta circles) or both self and other (overlap) tested for different object pairs. (i) Numbers of choice-coding neurons reported in the main text (GLM5, Equation 7) for the regressor ‘Object 3 chosen – Object 4 chosen’; these were the critical objects for observational learning as the partner chose them at session start while the recorded monkey chose them after object switch. (ii) Numbers of choice-coding neurons for the regressor ‘Object 1 chosen – Object 2 chosen’ (GLM5, Equation 7); these objects were initially chosen by the recorded monkey while the partner monkey chose them after object switch. For both regressors, the proportions of separate choice coding for self and other were significantly higher than those for joint choice coding (z-test for dependent samples; regressor ‘Object 3 chosen – Object 4 chosen’, self only versus joint: z = 3.48, p = 0.0005, other only versus joint: z = 4.13, p = 0.00003, self only versus other only: z = 0.73, p = 0.467; regressor ‘Object 1 chosen – Object 2 chosen’, self only versus joint: z = 4.03, p = 0.00005, other only versus joint: z = 2.65, p = 0.0079, self only versus other only: z = 1.48, p = 0.138). (iii) Numbers of choice-coding neurons specifically for the task period before object switch, which was the relevant task period for observational learning when the animals were choosing from different picture sets (GLM5, Equation 7): recorded monkey’s choices were modeled by the regressor ‘Object 1 chosen – Object 2 chosen’ whereas partner’s choices were modeled by the regressor ‘Object 3 chosen – Object 4 chosen’. The proportions of separate choice-coding for self and other were significantly higher than those for joint choice coding (z-test for dependent samples; self only versus joint: z = 2.65, p = 0.0079, other only versus joint: z = 2.65, p = 0.0079, self only versus other only: z = 0, p = 1.0). (iv) Numbers of choice-coding neurons for object-independent, order-referenced choices (regressor ‘First object chosen’, GLM5, Equation 7). The proportions of separate choice coding for self and other were significantly higher than those for joint choice coding (z-test for dependent samples; self only versus joint: z = 3.77, p = 0.0001, other only versus joint: z = 2.75, p = 0.006, self only versus other only: z = 1.15, p = 0.248). Taken together, these results confirmed that separate choice coding for partner and recorded monkey was significantly more prevalent in amygdala neurons than joint choice coding.

**Figure 4:**
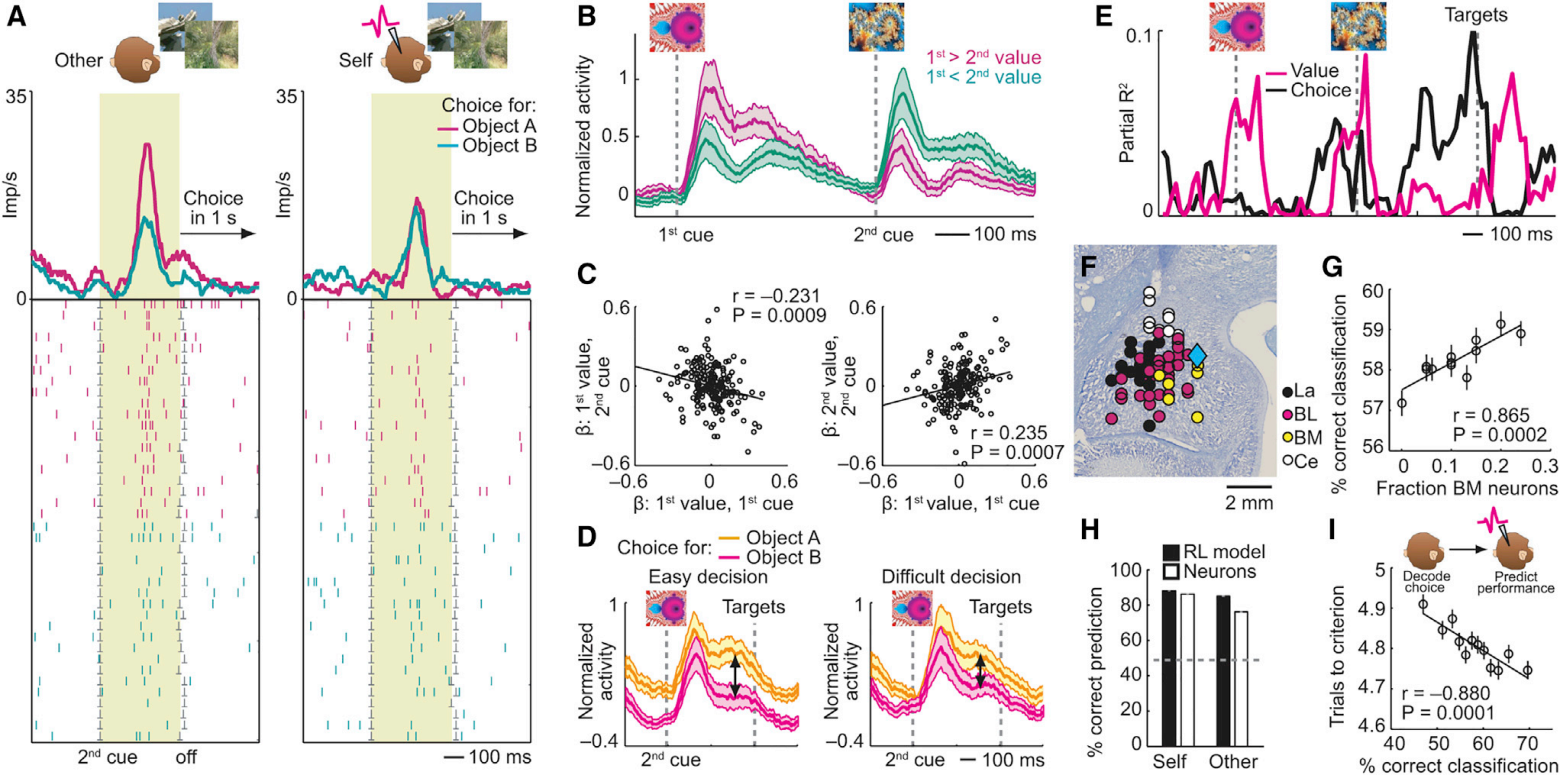
Amygdala Neurons Simulate the Partner’s Decision Making through Value Comparisons and Value-to-Choice Conversions (A) Single neuron predicting choice for partner but not recorded monkey. Responses to second object on partner’s (left) and recorded monkey’s trials (right), sorted by forthcoming object choice are shown. Recorded monkey was required to fixate objects without looking at partner. Monkeys could plan object choice but not left-right action before target appearance. (B) Neuronal value comparison on partner’s trials. Population activity of value-coding neurons during sequential presentation of partner’s choice objects. (C) Neuronal value slopes on partner’s trials indicated mutual-inhibitory value comparison. Left: anti-correlated value slopes for first object at first cue and second cue; object-value signals changed sign from first to second cue. Right: correlated values slopes for first and second object. (One data point is outside plotted range.) (D) Stronger choice signals for easier decisions. Population activity of choice-predictive neurons on partner’s trials for easy and difficult decisions (median-split by unsigned value difference) is shown. (E) Value-to-choice conversion before partner’s choice in a single neuron. Explained variance of value and choice regressors from sliding-window regression is shown. Activity transitioned from coding value (decision input, magenta) to predicting partner’s choice (decision output, black). (F) Location of neurons predicting partner’s choices. Diamond: neuron from (A). (G) Accuracy of neuronal decision simulation depended on basomedial neurons. (H) Neuronal choice prediction from classifier approximated reinforcement learning (RL) model. (I) Neuronal choice prediction during observational learning (before object switch) predicted recorded monkey’s post-switch performance. All averages are mean ± SEM. p values indicate Pearson correlation. See also Figures S3, S4, and S5.

Decision-making involves two stages: value inputs are compared between options and then converted to a choice output. Individual amygdala neurons dynamically coded these decision components during the recorded monkey’s trials to signal the monkey’s own choices (Figure S3), complying with computational decision theories (Deco et al., 2013, Wang, 2008). Surprisingly, the same decision-related activities occurred spontaneously before partner’s choices but in separate neurons, as if these neurons simulated the partner’s decision-making (Figure 4, described next). We refer to these neurons as simulation neurons because they dynamically coded key signatures of decision computation during social observation, in the absence of decision requirements for the recorded monkey.

The neuron in Figure 4A signaled the partner’s forthcoming object choice well before the partner’s observable action (p = 0.0009, multiple regression, Equation 7; Table S3); it failed to signal the recorded monkey’s own object choice. This neuron thus specifically encoded the predicted output of the partner’s decision process. Multiple regression identified neurons with activity related to partner’s choices, controlling for value and other covariates (31/205 neurons, 15%; p < 0.05, Equation 7). Importantly, separate neurons signaled the recorded monkey’s own choices for the same objects (37/205, 18%), whereas few individual neurons signaled object choices for both animals (9/205, 4%). Such separate choice coding for self and other in single neurons was significantly more prevalent than joint choice coding (z-test for dependent samples, p < 0.0005; Figure S3G). The distinct coding in separate neurons indicated that choice signals on partner’s trials did not simply reflect generalized, cue-evoked decision activity or erroneous decision preparation by the recorded monkey. Importantly, the animals did not mistake partner’s trials for their own (0.3% erroneous action attempts on partner’s trials). Thus, distinct amygdala neurons encoded the partner monkey’s predicted decisions.

In addition to predicting partner’s object choices, amygdala neurons encoded abstract choices in an order-based frame of reference, by signaling whether the partner would choose the first or second object on a given trial (Equation 7; Table S3). Again, separate neurons encoded order-referenced choices for partner and recorded monkey (Figure S3G). Notably, order-referenced choices were purely internal variables without explicit correspondence to sensory task events, which confirmed coding of internally simulated decisions.

**Figure S4:**
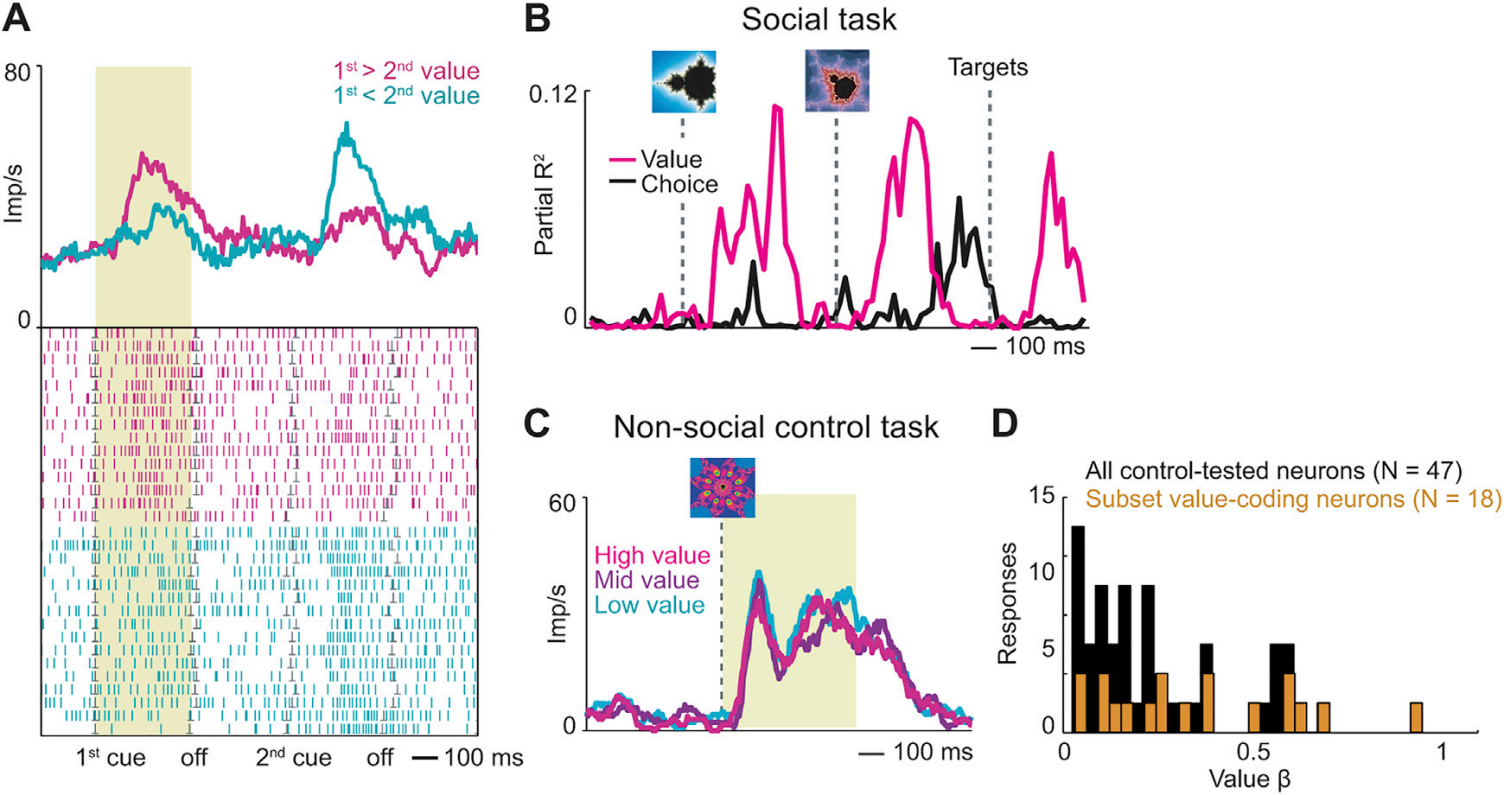
Value-Comparison Signals in Social Task and Relative Absence of Value Signals in Non-social Control Task, Related to Figure 4 **A,** Neuronal value comparison on partner’s trials in a single neuron. Activity of one value-coding neuron during sequential presentation of partner’s choice objects, sorted according to value of first object. **B, C,** Comparison between social observational learning task and non-social control task. **B,** Single neuron encoding value-to-choice transition on partner’s trials in social task. **C,** Activity of the same neuron recorded in a non-social control task. The neuron failed to signal value (reward probability) of conditioned stimuli that predicted reward for the recorded monkey in absence of social partner. **D,** Value coding in non-social control task. Histogram of value slopes (β) for 47 responses with significant value coding on other’s trials (Equation 7) that were tested in the non-social control task (black) and subset of 18 neurons (38%) with common significant value coding across tasks (orange).

Amygdala neurons seemed to construct predictions of partner’s choices by the same mechanisms used for the recorded monkey’s own choices, as indicated by the encoding of three key decision-making signatures. First, amygdala neurons dynamically encoded value comparisons of partner’s choice options, as shown by responses to sequential objects that depended on both objects’ values (Figure 4B, 86/205 neurons, Equation 7; cf. Figure S3). Specifically, neurons signaled values for partner’s competing choice objects with anti-correlated slopes (Figure 4C). Competing objects thus had opposing influences on neuronal activity—a characteristic signature of value comparison by mutual inhibition (Deco et al., 2013, Wang, 2008). Neurons encoding such value comparisons on partner’s trials often failed to code value in a non-social control task, performed separately from the main task (29/47 control-tested neurons, 62%, Figure S4); these neurons thus did not reflect generalized, cue-evoked valuation. Second, decision signals on partner’s trials were stronger for easier decisions (i.e., larger value differences, Figure 4D), resembling decision neurons in non-social tasks (Kim and Shadlen, 1999). Stronger choice signals for easier decisions are consistent with the resolution of an underlying mutual-inhibitory winner-take-all competition, whereby neurons representing the “winning” choice option show higher activity for easier, clearly resolved value comparisons (Deco et al., 2013, Wang, 2008). Third, in individual neurons, sequential value signals on partner’s trials evolved into explicit predictions of partner’s choices (“value-to-choice conversions”; Figure 4E, 22/205 neurons; 11%). These neurons thus encoded the complete information-processing sequence involving value comparison and choice prediction, indicative of a neuronal decision process. Different from object-value neurons, which particularly involved lateral nucleus, neurons with simulation-related activities were especially (but not exclusively) linked to basomedial nucleus, which encoded partner’s value-to-choice conversions more accurately and more frequently than lateral neurons (p = 8.2 × 10^−4^, χ^2^ test, Figures 4F and 4G). Notably, neurons selective for partner’s choices (Figure 4F) were closely intermingled with those selective for recorded monkey’s choices (Figure S3F).

Taken together, our notion of neuronal decision simulation derived from partner-specific choice predictions and computationally well-characterized signatures of an underlying decision process, including dynamic value comparisons, sensitivity to choice difficulty, and explicit value-to-choice conversions. Beyond choice prediction, individual simulation neurons encoded some or all of these formal decision-making signatures on partner’s trials, well before the partner’s observable action.

### Neuronal Decision Simulations Are Related to a Monkey’s Own Learning Success and Observation of the Partner’s Actions

Using population decoding, we tested relationships between neuronal decision signals and behavior. Support-vector-machine decoding from unselected neurons showed that neuronal coding of partner’s and recorded monkey’s decisions was nearly optimal: its accuracy matched reinforcement models that derived choice predictions from reward-choice history (Figure 4H). Across sessions, the accuracy with which neurons encoded partner’s decisions predicted the recorded monkey’s observation-learning success, with better performance after more accurate decision coding (Figure 4I, p = 1.3 × 10^−6^, partial correlation controlling for value coding, cf. Figure 3K). This behavioral relationship was found for activities measured during object presentation before partner’s action; it therefore demonstrated the importance of internal decision simulations for observation learning. In addition, choice-decoding accuracy during action observation (when the partner reached for the chosen object) also reflected learning performance (p = 1.1 × 10^−5^). Performance relationships were not found when decoding control variables, such as whether self or partner was choosing (p = 0.296).

**Figure S5:**
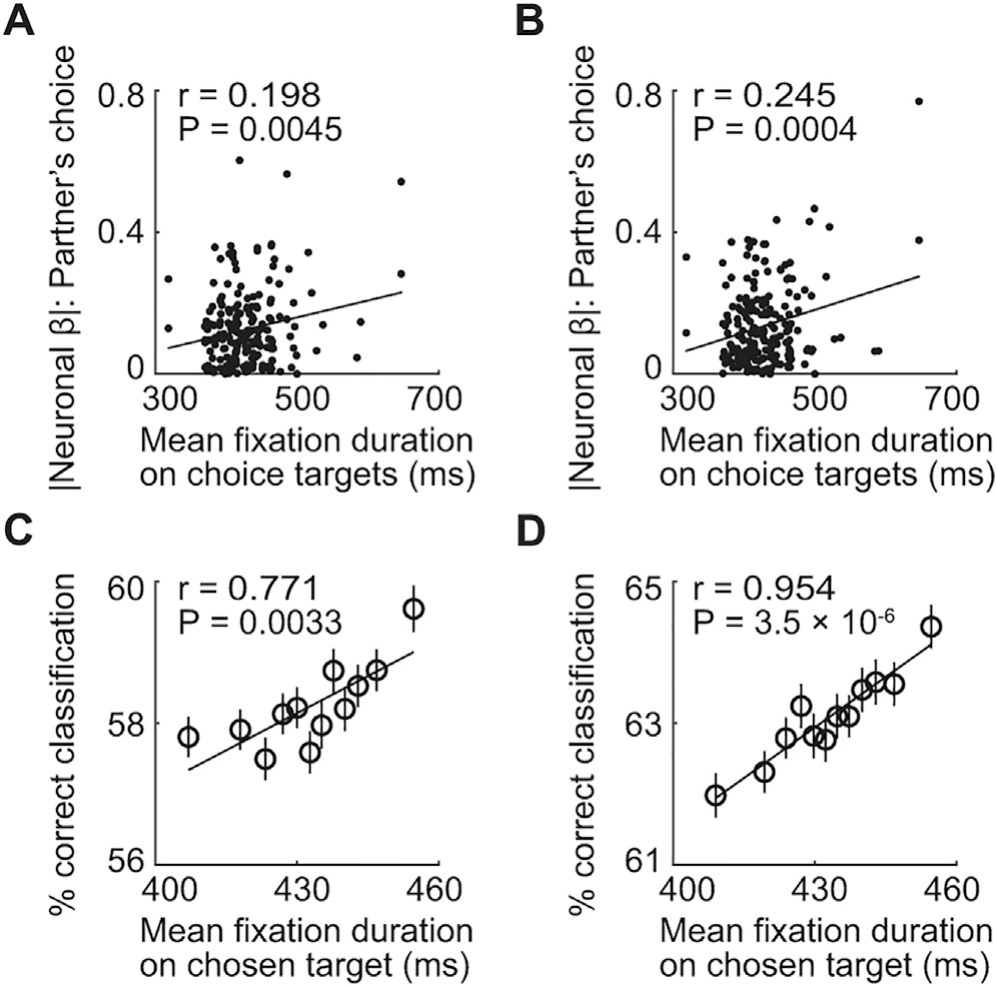
Relationships between Simulation Neurons and Observation of Partner’s Choices, Related to Figure 4 **A,** Neuronal value slopes (unsigned βs) of simulation neurons plotted against the time that the recorded monkey spent looking at partner’s choice objects when the partner executed the choice. Neuronal βs were obtained from Equation 7 and correspond to single-neuron sensitivities to partner’s forthcoming choice, measured before partner’s action (N = 205 neurons, across-neuron analysis). Looking durations were measured as the mean duration of the recorded monkey’s fixations, within a recording session, that fell onto the touch screen area corresponding to partner’s choice objects and action targets (*cf.*Figure 1A) in the period from onset of choice-cues in left-right arrangement until onset of reward receipt. The relationship was also significant for fixations specifically of partner’s chosen object (r = 0.152, p = 0.0299); as a control, the relationship was not found for neuronal encoding of recorded monkey’s own choices (p = 0.563), or for fixations of partner’s face during action period (p = 0.651) or reward period (p = 0.155). **B,** Neuronal value slopes for simulation neurons encoding partner’s abstract choices in an order-based frame of reference (choice of first versus second object), plotted against fixation durations as in (A). **C,** Relationship between neuronal decoding accuracy of partner’s forthcoming choices and fixation durations. Leave-one-out cross-validated accuracy of support-vector-machine classifier decoding partner’s choices from randomly sampled subsets of 20 neurons (5,000 iterations), plotted against mean fixation duration of partner’s chosen objects. Neuronal activity used for classification was from the period before objects switched between animals and was measured on each trial during sequential object presentation, i.e., before partner’s action (choice prediction). Fixations were measured during action period as in (A). **D,** Relationship between neuronal decoding accuracy of partner’s observed choices and fixation durations. Same analysis as in (C) but neuronal choice decoding was performed during the period when the partner executed the choice (choice observation).

We examined whether simulation activities depended on the recorded monkey’s observations of partner’s choices. Across sessions, single-neuron sensitivities to partner’s forthcoming choices reflected the amount of time that the recorded monkey spent looking at the partner’s choices when the partner reached for choice targets on the touch screen (Figures S5A and S5B). Neuronal population decoding of partner’s choices was also more accurate in sessions in which the recorded monkey spent more time observing the partner’s choices (Figures S5C and S5D).

Thus, neuronal coding of partner’s simulated decisions was linked to the recorded monkey's own performance and to observation of partner’s choices. Naturally, any simulation activity would need to be informed by observations of partner’s previous choices; however, simulation activity within trials preceded the partner’s observable choice and thus did not reflect simple observation.

### Population Codes for Value and Choice in Amygdala Subnuclei

**Figure 5:**
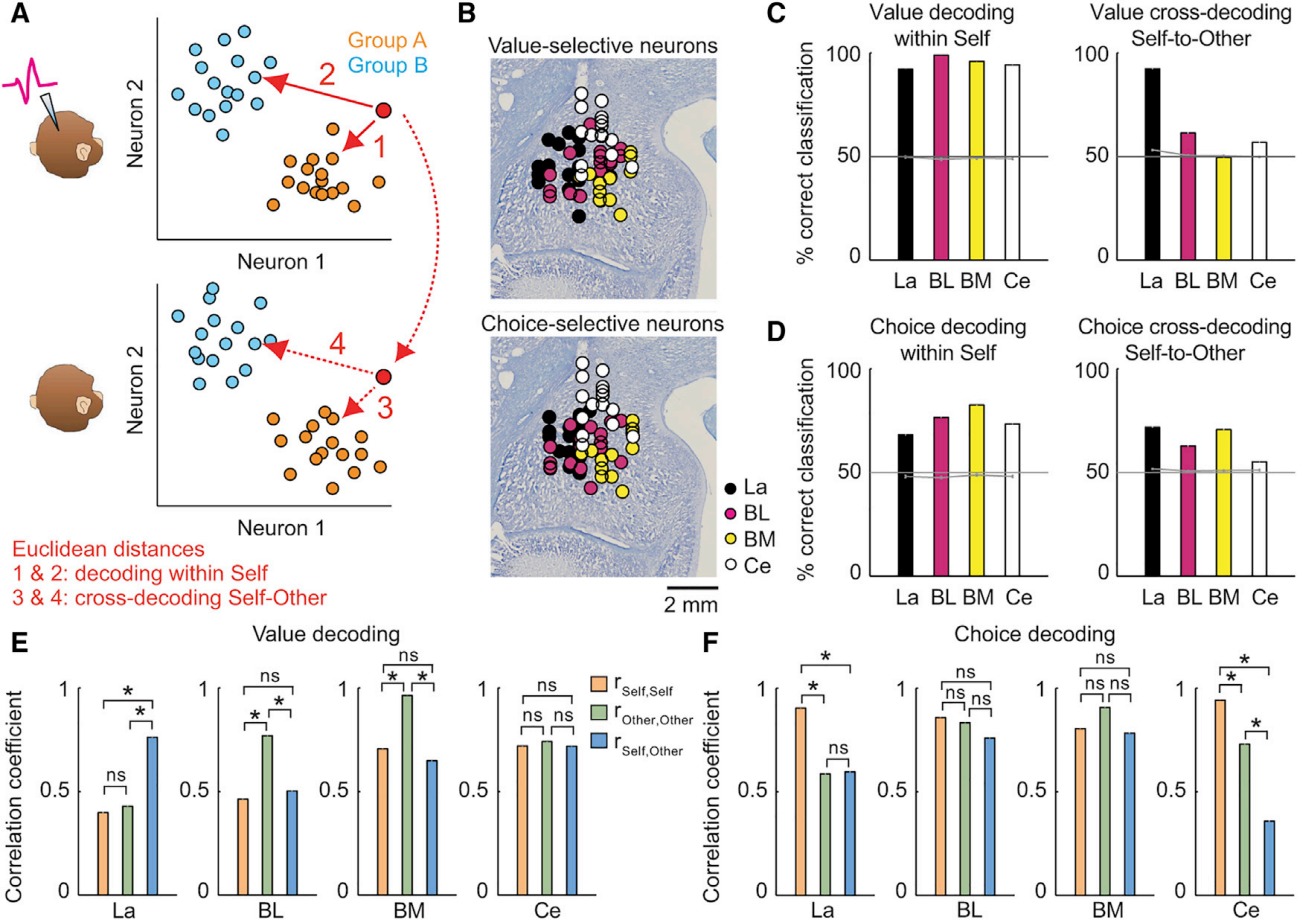
Population Codes for Value and Choice in Amygdala Subnuclei (A) Decoding approach. A nearest-neighbor classifier computed Euclidean distances between single-trial activity vectors (red point) and mean activity vectors for different value levels or choices (groups A, B) measured on recorded monkey’s trials (decoding within Self, top panel). Cross-decoding used mean activity vectors from partner’s trials (cross-decoding Self-Other, bottom panel). (B) Location of value-coding neurons and choice-coding neurons for decoding (20 neurons per nucleus, selected based on regression coefficients; collapsing anterior-posterior levels resulted in display-overlap of nuclei). (C) Value-decoding accuracies for subnuclei within self (left) and cross-decoding (right) before objects switched between animals. Gray line: chance. (D) Choice-decoding accuracies for subnuclei. (E) Correlations between mean activity vectors related to different value levels. r_Self,Self_, activity vectors for different values within-self; r_Other,Other_, correlation for different values within-other; r_Self,Other_, correlation for same value levels across animals. (F) Correlations between mean activity vectors related to different choices. ^∗^Significantly different correlations (p < 0.005, Bonferroni corrected). Error bars indicate SEM.

We adapted a biologically plausible nearest-neighbor classifier to examine coding differences between amygdala subnuclei (Figures 5A and 5B). Value decoding and choice decoding from mean activity vectors was accurate across nuclei, with higher accuracies for value decoding (Figures 5C and 5D). We examined the capacity for transferring value and choice signals between self and other, by classifying single-trial activity vectors measured on recorded monkey’s trials using mean activity vectors from partner’s trials. Cross-decoding for value was accurate in lateral nucleus but declined sharply in other nuclei (Figure 5C). By contrast, cross-decoding for choice was above chance but overall less accurate than value cross-decoding, involving lateral, basomedial, and basolateral nucleus (Figure 5D). Thus, lateral nucleus was particularly important for value cross-decoding, as also suggested by separate support-vector-machine classification (cf. Figure 3G).

To examine these codes more directly, we computed correlations between mean-activity vectors. In lateral nucleus, correlations between population vectors for different value levels were low within-self and within-other (Figure 5E), indicating appropriate separation of different values; by contrast, activity vectors for identical value levels were highly correlated between self and other (Figure 5E), significantly more so than in other nuclei (p < 0.001). Thus, lateral-nucleus neurons responded similarly to self and other’s high-value objects, suggesting a shared value code suitable for cross-decoding. Self-other cross-correlations for choices were significantly lower than or not significantly different from within-animal correlations (Figure 5F).

Thus, biologically plausible decoding from small groups of selected neurons suggested a shared value code between self and other, specifically in lateral nucleus.

### Amygdala Neurons Discriminate Self-Trials from Other-Trials

**Figure 6:**
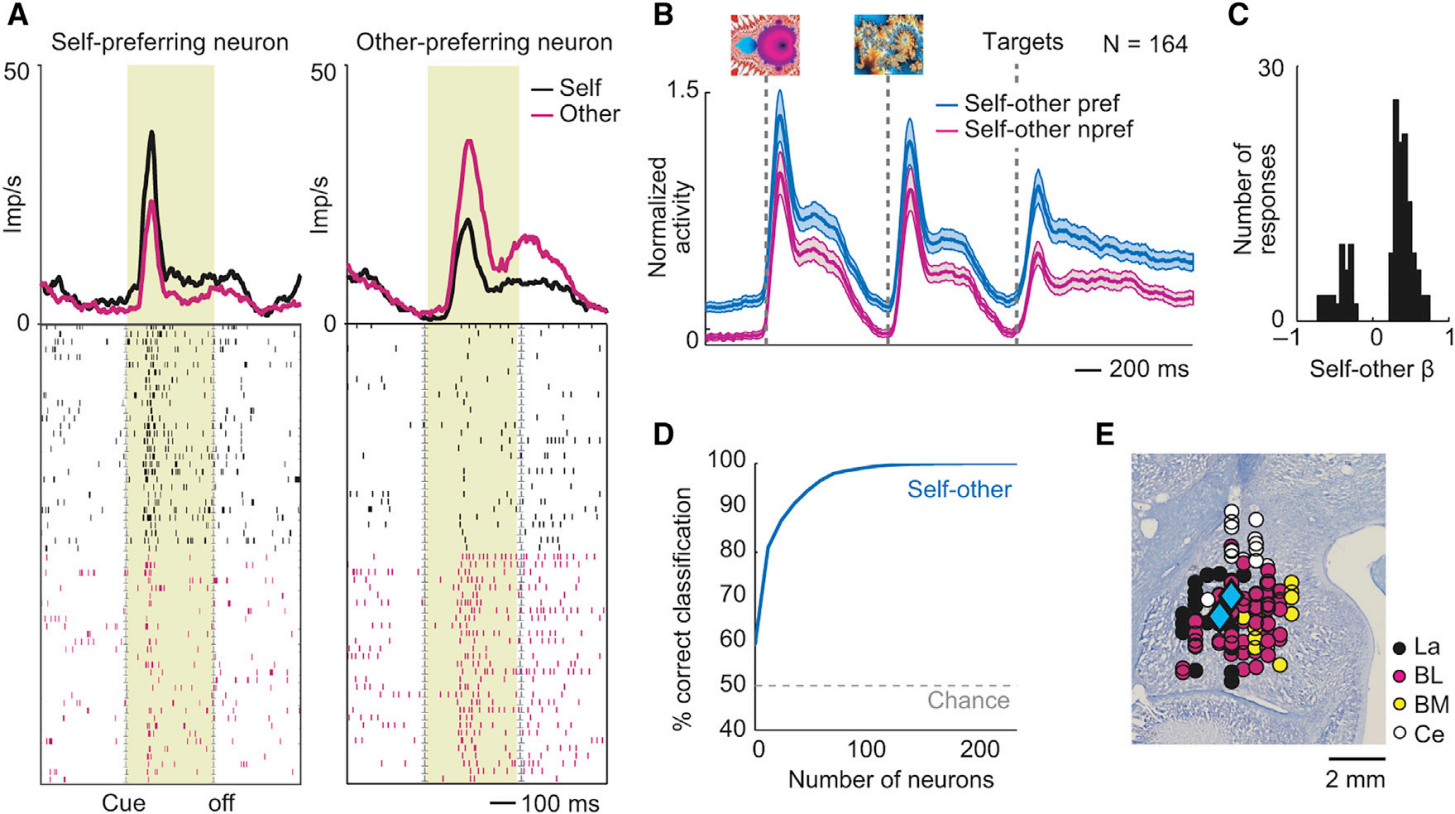
Social Self-Other Neurons (A) Two amygdala neurons differentiating self and other trials. Left: neuron with stronger activity during presentation of second choice cue on recorded monkey’s trials, compared to partner’s trials. Right: neuron with stronger activity on partner’s trials. (B) Population activity of self-other discriminating neurons. Activity was sorted by each neuron’s preferred trial type (self versus other). (C) Histogram of regression coefficients of neurons with self-other coding. (D) Support-vector-machine decoding accuracy of self versus other trials from neuronal activity. (E) Locations of self-other-coding neurons. Diamonds: neurons from (A). All averages are mean ± SEM.

In social situations, self-other discrimination is crucial for agent-specific neural reference frames (Chang, 2017, Wittmann et al., 2016). We found that many amygdala neurons showed differential activity on self-trials and other-trials (164/205 neurons, 80%; p < 0.05, Equation 8; Table S4). The neuron in Figure 6A (left) showed increased activity during object presentation when it was the recorded monkey’s turn to choose, while the neuron in Figure 6A (right) showed increased activity when it was the partner’s turn. Self-other signals occurred throughout trial epochs (Figure 6B). They were not explained by object value, choice, or other factors, which were regression covariates (Figure 6C). Amygdala population activity enabled highly accurate self-other discrimination (Figure 6D). Self-other discriminating neurons were prevalent across amygdala subnuclei (Figure 6E).

### Computational Model of Separate Amygdala Decision Circuits for Self and Other

**Figure 7:**
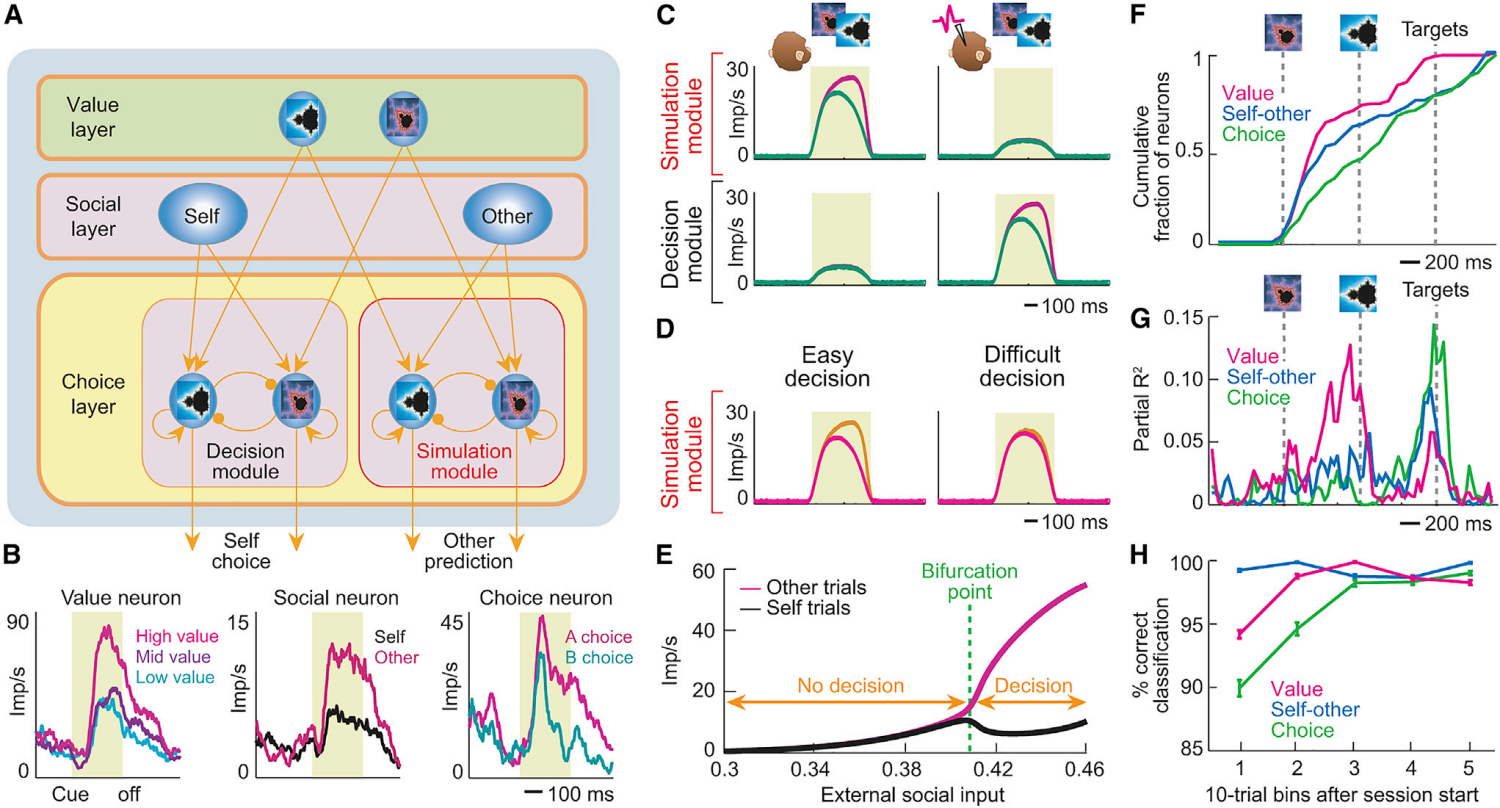
Biophysically Plausible Model of Amygdala Circuits for Social Decision Simulation (A) Model architecture inspired by recorded neuron types. Object-specific value neurons (Value layer) and self-other discriminating social neurons (Social layer) provide convergent excitatory inputs to two separate decision systems (Choice layer) for computing recorded monkey’s choices (Decision module) and for simulating partner’s choices (Simulation module). Within each choice-layer module, pools of object-specific neurons with recurrent excitatory connections implement decision-making by mutual-inhibitory winner-take-all competition (mediated by interneurons, not shown). Depending on self-other bias, value inputs initiate competition selectively in one of the two choice-layer modules; once competition is resolved, the winning pool enters a high-activity attractor state that represents the choice. (B) Neuron types on which model is based. Three representative recorded example neurons are shown. (C) Modeled choice-layer neurons. Neurons in simulation module signal partner’s choice on partner’s trials when social layer provides other bias (top left) but not on recorded monkey’s trials when social layer provides self bias (top right). Conversely, neurons in decision module signal choice for recorded monkey with self bias (bottom right) but not other bias (bottom left). (D) Model reproduces decision-difficulty effect of simulation neurons. (E) Bifurcation diagram of model activity. Differential, choice-predictive activity of neurons in simulation module depends on strength of self-other input from social layer. (F) Latencies of signals for value, self-other, choice across recorded neurons. Cumulative fraction of significant neurons following cue onset. Value and self-other signals preceded choice signals (p < 0.05, Wilcoxon test). (G) A single recorded amygdala neuron integrating value, social, and choice information. Explained variances from sliding-window regression are shown. (H) Neuronal discrimination of self from other’s choices increased within session. Decoding accuracy for high-low value, self-other trials, and self-choice versus other-choice were calculated from recorded neuronal responses within ten-trial windows over two-trial steps. All averages are mean ± SEM. See also Figure S6.

From these data, we hypothesized that separate decision systems in primate amygdala might compute own choices and simulate choices of social partners. We designed an attractor neural-network model (Figure 7A) in which distinct pools of decision neurons generate choices for self and other (“choice layer,” cf. Figure 4A), based on conjunctive inputs from shared object-value neurons (“value layer,” cf*.* Figure 3B), and self-other discriminating “social neurons” (“social layer,” cf. Figure 6A). By biasing the choice layer, social neurons selectively enable value-based decision-making in one of two separate systems. Further examples of these recorded functional neuron types are shown in Figure 7B. These neuron types were frequently observed: 127 object-value neurons (62%), 37 self-choice neurons (18%), 31 other-choice neurons (15%), 164 self-other neurons (80%), with individual neurons often integrating these signals.

We constructed a biologically plausible implementation of this circuit architecture (see STAR Methods) in which different choice-selective neuronal populations compete with each other to implement decision-making through mutual inhibition in an attractor neural network (Wong and Wang, 2006). Our approach followed previous studies that linked decision computations to anti-correlated neuronal value slopes (Strait et al., 2014) and value-to-choice conversions in single neurons (Grabenhorst et al., 2012, Tsutsui et al., 2016). These approaches are based on previous demonstrations that such analyses can be linked to mutual inhibition processes in attractor models (Chau et al., 2014, Hunt et al., 2012).

The model reproduced our main data features as follows. Given constant value inputs, biasing from the social layer’s “other” neurons generated choice signals only in the other (simulation) decision system but not in the self decision system (Figure 7C), with stronger signals for easier decisions (Figure 7D). Conversely, biasing from self neurons generated choice only in the self system. Selective decision computation critically depended on the social layer’s input strength (Figure 7E): value inputs alone were insufficient to drive a decision computation; rather, the simulation module required additional activation by appropriate input from self-other neurons to show differential, choice-predictive activity. Data from recorded amygdala neurons further supported the model’s plausibility: population coding latencies conformed to the model’s implied information flow (Figure 7F), whereby value signals and self-other signals evolve into choice signals. Moreover, individual amygdala neurons combined the model’s key signals (Figure 7G, 20/205 neurons, 10%).

**Figure S6:**
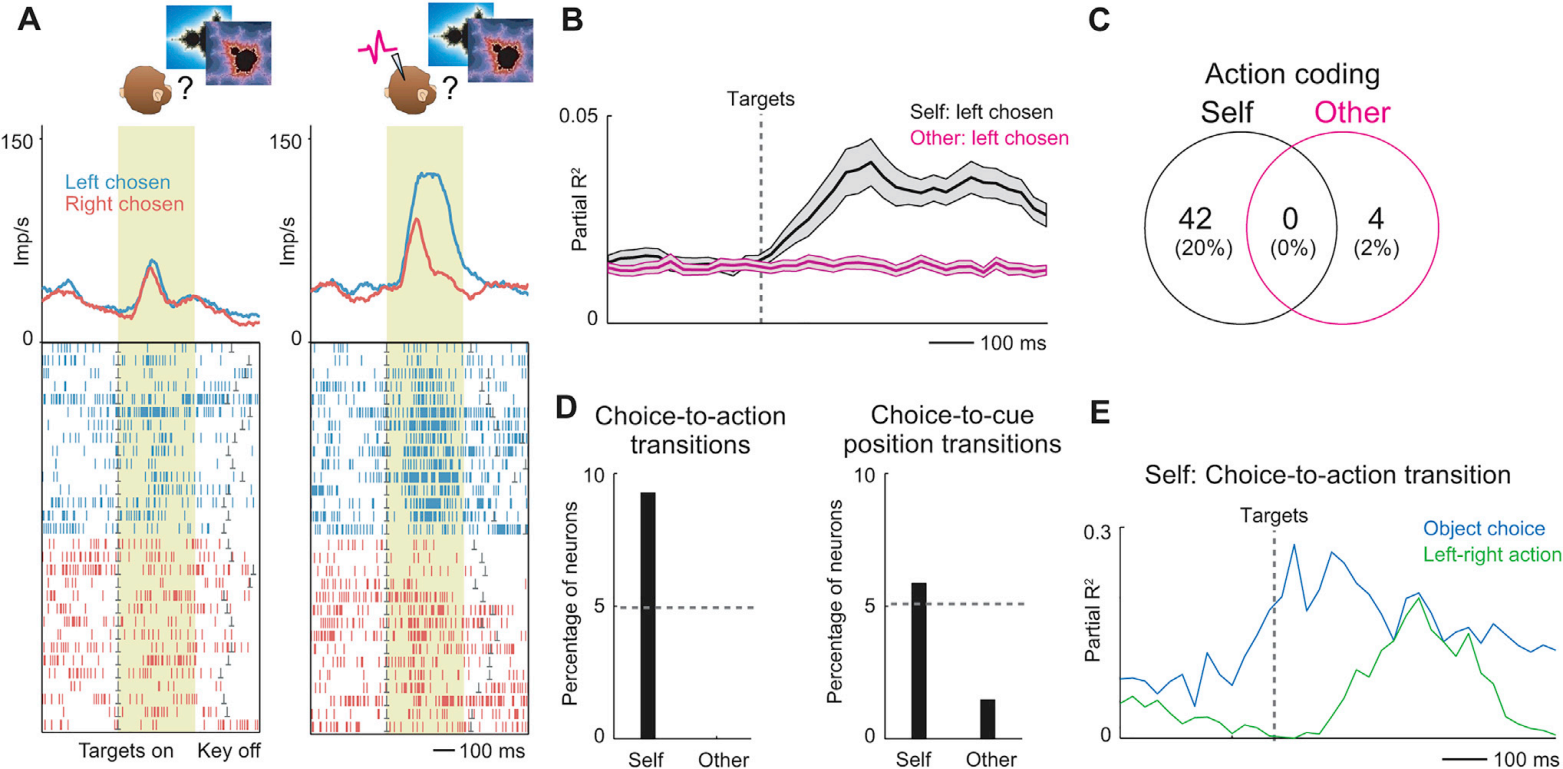
Amygdala Neurons Encode Action Information for Recorded Monkey but Not for Partner, Related to Figure 7 **A,** Single neuron signaling recorded monkey’s left versus right arm movements during target presentation. The neuron responded more strongly when the recorded monkey performed an arm movement toward a touch target on the left side of the monitor compared to movement toward the right side. The neuron failed to distinguish observed left-right actions on partner’s trials. **B,** Population data across all recorded neurons. Explained variance by left-right action regressor during target presentation (sliding-window regression). Significant information about left-right actions was encoded by neurons on recorded monkey’s trials (black) but not on partner’s trials (magenta). **C,** Action coding for recorded monkey and partner. A significant number of neurons signaled recorded monkey’s executed actions; by contrast, few neurons signaled observed partner’s actions and no neuron jointly signaled partner’s and recorded monkey’s actions. **D,** Choice-to-action transitions. Left: Some neurons dynamically encoded the recorded monkey’s object choice before encoding the recorded monkey’s action. Such choice-to-action transitions were entirely absent on partner’s trials. Right: Corresponding data for neurons transition from choice-coding to coding of left-right cue position. **E,** Choice-to-action-transition in a single amygdala neuron. On recorded monkey’s trials, the neuron signaled the monkey’s forthcoming object choice just before onset of choice targets. Following target presentation, the neuron’s activity began to signal the monkey’s left-right action. All averages are mean ± s.e.m.

We propose that distinct simulation neurons emerge naturally through a self-organization process, by learning to respond to coactive object-value neurons and social other neurons. Supporting this idea, neuronal separation of partner’s from recorded monkey’s choices increased over time (Figure 7H). Notably, while some neurons linked choice signals to action signals for the recorded monkey (21/205 neurons; Figure S6, Equation 10; Table S5), such choice-to-action transitions were entirely absent for the partner (0/205 neurons). This result could suggest that, while one decision circuit computes the recorded monkey’s own choices to guide actions, a distinct decision circuit implements offline simulations of the partner’s decisions without translation into action.

These results support our hypothesis that separate decision systems in amygdala compute choices for self and social others. Amygdala inhibitory circuits and plasticity mechanisms (Janak and Tye, 2015, Pape and Pare, 2010) seem potentially suited for implementing this design.

## Discussion

These data show that when monkeys observe and learn from each other’s choices, amygdala neurons derive object-specific reward values from social observation, and dynamically convert these values to representations of the partner’s forthcoming choices. Neuronal object values were subjective, as they reflected partner’s and recorded monkey’s distinct reinforcement histories (Figures 3B and S2). Neurons signaled values from observation and experience in a common code that was highly accurate and transferable between self and other, particularly in lateral nucleus (Figures 3G–3J and 5). Such common, object-centric value coding facilitates reward learning irrespective of who is choosing and provides versatile inputs for own decisions and social simulations. Accordingly, the recorded monkey learned better from more accurately coded values (Figure 3K). By processing partner’s and recorded monkey’s values in a shared code, these amygdala neurons provide a physiological basis for integrating own and others’ experiences.

In contrast to object-referenced values, amygdala neurons encoded decisions with a social reference frame: specific simulation neurons signaled partner’s predicted choices distinct from recorded monkey’s own choices (Figure 4A). These neurons encoded three key signatures of the simulation of partner’s decision-making, including sequential value comparisons, sensitivity to decision difficulty and explicit value-to-choice conversions (Figures 4B–4E). The simulation activities unfolded dynamically and spontaneously, well before partner’s observable choices and without decision requirement for the recorded monkey, resulting in accurate predictions of partner’s choices. Amygdala neurons thus seemed to construct partner’s choice predictions by the same mechanisms as those underlying the recorded monkey’s own choices (cf. Figure S3), typical of mental simulation (Adolphs, 2006, Gordon, 1996, Shanton and Goldman, 2010). Simulating others’ decisions with dedicated neurons is unanticipated by cognitive theories but functionally crucial: it enables offline simulations that prevent erroneous acting-out of other’s choices—shown by absent choice-to-action conversions for partner (Figure S6)—and disambiguates other’s from own choice signals for downstream processing.

Although population decoding showed some capacity for self-other cross-transfer of choice codes (Figure 5D), this likely resulted from pooling individual neurons with selective choice coding for self or other. This result and the anatomical intermingling of neurons coding own or other’s choices suggest that simulation neurons may be difficult to detect in human imaging, which averages over neural populations.

We constructed a biophysically plausible attractor neural-network model based on the functional neuron types we recorded (Figures 7A and 7B). The model captured key data features, including selective decision computation for self or other and suggested that simulation neurons can emerge from conjunctive object-value and self-other signals (Figures 7C–7H). These results are consistent with the existence of separate decision circuits in primate amygdala that use common object values to compute own decisions and simulate decisions of social partners. We suggest that the mapping from object-centric value neurons to separate decision neurons for self and other could involve a competitive, feature-detection network (Rolls and Treves, 1998) coupled to an attractor decision-making network. When confronted with novel choice objects, this competitive network would learn, via lateral or mutual inhibition, to respond to repeatedly co-active object-selective and self-other neurons, and subsequently activate different choice neurons for self and other. This suggestion for how simulation neurons “emerge” (i.e., are functionally set up) via self-organization is supported by the observed gradual separation of self-choice and other-choice signals (Figure 7H).

Previous research identified important building blocks for primate social behavior in amygdala, including face neurons (Gothard et al., 2007, Leonard et al., 1985, Rutishauser et al., 2013). In a classic study, face neurons were prevalent in basomedial (accessory basal) nucleus (Leonard et al., 1985), which we found particularly implicated in decision simulations (Figure 4G). Recent studies described amygdala neurons coding facial expression (Gothard et al., 2007), reward expectations for others (Chang et al., 2015), and conspecifics’ hierarchical rank (Munuera et al., 2018). Simulation neurons could provide an output channel for these signals by locally integrating them to choice predictions for specific social partners. Notably, the distinct choice coding for self or other and the dynamic value-to-choice conversions suggest that simulation activities did not reflect reward expectation or state valuation (Belova et al., 2008).

In a previous study, amygdala neurons’ value sensitivities were correlated between self and other, but only when values were behaviorally relevant for the recorded monkey (Chang et al., 2015). In the present study, amygdala value codes were transferable between self and other, but mainly when objects were relevant for observational learning (Figures 3J, inset, and 5C). Thus, amygdala neurons encode object values especially when they are behaviorally or socially relevant. Previous studies also showed neuronal discrimination between biological partners and computer opponents (Báez-Mendoza et al., 2013, Haroush and Williams, 2015, Hosokawa and Watanabe, 2012). It will be interesting to test how simulation neurons respond to computer opponents.

A recent study first reported neurons in cingulate cortex that predicted other’s forthcoming decisions in a strategic game (Haroush and Williams, 2015). Our study builds on this critical work by identifying the neuronal value inputs and decision computations underlying such choice predictions, and their potential origin in attractor neural networks. Determining whether social predictions first arise in cingulate or amygdala would require simultaneous recordings. Nevertheless, amygdala neurons seem suited to construct decision simulations, as suggested by their value-to-choice transitions. Importantly, amygdala lesions affect encoding of stimulus-reward associations in cingulate cortex (Rudebeck et al., 2017), and both amygdala and cingulate participate in learning (Klavir et al., 2013) and prosocial choices (Chang et al., 2013b, Chang et al., 2015). Thus, it is likely that anterior cingulate cortex (Haroush and Williams, 2015) and amygdala (shown here) interact in modeling partners’ choices.

Our findings are distinct from social error monitoring and action observation. Neuronal responses to others’ performance errors are found in medial frontal cortex (Yoshida et al., 2012) and striatum (Báez-Mendoza and Schultz, 2016). Prefrontal neurons also track choices and outcomes during competition with opponents (Hosokawa and Watanabe, 2012, Lee and Seo, 2016, Seo et al., 2014). Such signals likely contribute to macaque observational learning shown here and previously (Subiaul et al., 2004). However, error signals are reactions to external events that follow partner’s choices, different from the predictive, purely internal decision simulations reported here.

The separate coding of other’s from own choices by distinct neurons and the prospective encoding of purely internal, unobservable value comparisons, value-to-choice conversions and choice predictions distinguishes simulation neurons from mirror neurons, which respond to both executed and observed actions (Fabbri-Destro and Rizzolatti, 2008). Importantly, the present choice-predictive signals were not sensory responses to the other’s choice, as they were measured during sequential cue presentation well before partner’s overt choice. At the time of partner’s action, we found no evidence for action-coding mirror neurons in amygdala (Figure S6).

Our findings link recent concepts on the functions of amygdala neurons in decision-making (Grabenhorst et al., 2012, Grabenhorst et al., 2016) to the amygdala’s well-known role in social behavior. Classical studies demonstrated deficient emotion recognition and social judgment in humans with amygdala lesions (Adolphs et al., 1998, Adolphs et al., 2005). Current accounts of these deficits emphasize the amygdala’s importance in social perception and in directing attention to specific face parts (Adolphs et al., 2005, Rutishauser et al., 2013, Rutishauser et al., 2015). Our data point toward an additional mechanism, whereby amygdala simulation neurons may actively reconstruct social partners’ mental states. A “constructive” neural mechanism for social cognition in amygdala had previously been proposed (Adolphs, 2006), but its single-neuron basis remained unclear. The simulation neurons described here seem well suited to support understanding of others’ mental states, as they translate observation-derived values into representations of other’s decisions.

The amygdala is implicated in autism and other conditions with atypical social cognition, including social anxiety (Amaral, 2002, Baron-Cohen et al., 2000, Lai et al., 2014, Rutishauser et al., 2013). Our data and model (cf. Figures 7A and 7B) may offer new insights into these conditions by specifying single-neuron building blocks and computational architectures for social cognition. We speculate that dysfunction or absence of amygdala simulation neurons, or their inputs, could impoverish social cognition by reducing an individual’s ability to simulate others’ mental states. Deficient neuronal simulation by the amygdala could play a role in the poor perspective-taking and social communication seen in autism. Conversely, hyperactivity of amygdala neurons might exaggerate spontaneous simulation of others’ mental processes. Given the amygdala’s outputs to emotional and physiological effector systems (Johansen et al., 2011), such exaggerated social simulations could provoke somatic symptoms typical of social anxiety (Amaral, 2002). Further, our model implies that altered connection-weights between neuron types (cf. Figure 7A) or instability in the simulation module’s attractor-dynamics could disrupt social cognition.

Our data and model suggest a neurobiological account of mental simulation as neural decision computation. The findings have implications for emerging directions in artificial intelligence in which machines are trained to model social partners’ minds (Rabinowitz et al., 2018). Based on single-neuron data, we propose a solution to this computational problem: convergence of value signals and self-other signals onto decision neurons enables the primate amygdala to alternately compute choices for self and other. Such flexibility in processing own and others’ mental states is crucial in primate life, which is governed by complex social hierarchies that affect amygdala structure and function (Noonan et al., 2014). The amygdala simulation neurons reported here could allow primates to reconstruct their social partner’s mental states and may constitute simple precursors for human mentalizing capacities, such as theory of mind.

## STAR★Methods

### Key Resources Table

**Table:** 

REAGENT or RESOURCE	SOURCE	IDENTIFIER
**Experimental Models: Organisms/Strains**		

Rhesus macaques (Macaca mulatta)	Centre for Macaques (CfM)	N/A

**Software and Algorithms**		

MATLAB	MathWorks	http://www.mathworks.com
Plexon offline sorter	Plexon	https://plexon.com

**Other**		

Microelectrodes	FHC	https://www.fh-co.com/

### Contact for Reagent and Resource Sharing

Further information and requests for reagents and resources should be directed to and will be fulfilled by the Lead Contact, Dr. Fabian Grabenhorst (fg292@cam.ac.uk).

### Experimental Model and Subject Details

All animal procedures conformed to US National Institutes of Health Guidelines. The work has been regulated, ethically reviewed and supervised by the following UK and University of Cambridge (UCam) institutions and individuals: UK Home Office, implementing the Animals (Scientific Procedures) Act 1986, Amendment Regulations 2012, and represented by the local UK Home Office Inspector; UK Animals in Science Committee; UCam Animal Welfare and Ethical Review Body (AWERB); UK National Centre for Replacement, Refinement and Reduction of Animal Experiments (NC3Rs); UCam Biomedical Service (UBS) Certificate Holder; UCam Welfare Officer; UCam Governance and Strategy Committee; UCam Named Veterinary Surgeon (NVS); UCam Named Animal Care and Welfare Officer (NACWO).

Three healthy adult male rhesus monkeys (Macaca mulatta) participated in the present experiments: two monkeys (weighing 10.5 and 12.3 kg) participated as recorded monkeys, and a third monkey (weighing 12.0 kg) participated as partner monkey. The animals had not been used for previous experiments. The number of animals used is typical for primate neurophysiology experiments. The animals were housed in groups of two or three animals; the three animals participating in the present study lived in different groups. At the time of neurophysiological recordings, the animals were highly trained in the experimental task.

### Method Details

#### Neurophysiological recordings

The experimental procedures for neurophysiological recordings from amygdala in awake, behaving macaque monkeys followed our previous studies (Grabenhorst et al., 2012). A head holder and recording chamber (Gray Matter Research) were fixed to the skull under general anesthesia and aseptic conditions. We used bone marks on coronal and sagittal radiographs to localize the anatomical position of the amygdala in reference to the stereotaxically implanted chamber, as described previously (Grabenhorst et al., 2012). Specifically, we located the amygdala posterior to the sphenoid bone, rostral to the posterior clinoid processes at and above the dorsoventral position of the posterior clinoid process. We recorded activity from single amygdala neurons from extracellular positions during task performance, using standard electrophysiological techniques including on-line visualization and threshold discrimination of neuronal impulses on oscilloscopes. We aimed to record representative neuronal samples from the dorsal, lateral, and basal amygdala. A stainless steel tube (0.56 mm outer diameter) guided a single tungsten microelectrode of 0.125 mm diameter and 1- to 5-MΩ impedance (FHC Inc.) through the dura and assured good targeting of subcortical structures. A hydraulic micromanipulator (MO-90; Narishige, Tokyo, Japan) served to advance the microelectrode vertically in the stereotaxic plane. Neuronal signals were amplified, bandpass filtered (300 Hz to 3 kHz), and monitored online with oscilloscopes. Somatodendritic discharges from single amygdala neurons were distinguished from background noise and other neurons using a time threshold window discriminator (WD-95; Bak Instruments), which produced a 1.0-ms-long standard transistor-transistor logic (TTL) pulse for each neuronal impulse that helped in the online inspection of neuronal recordings. Behavioral data, digital signals from the impulse window discriminator, and analog eye position data were sampled at 2 kHz on a laboratory computer with custom MATLAB (Mathworks Inc.) code. We recorded analog impulse waveforms at 22 kHz and sorted them offline for data analysis, using cluster-cutting and principal component analysis (Offline sorter; Plexon).

During recordings, we sampled activity from about 400 amygdala neurons and recorded and saved the activity of neurons that appeared to respond to any task event during online inspection of several trials. Thus, we aimed to identify task-responsive neurons but we did not preselect based on more specific response characteristics. This procedure resulted in a database of 205 neurons which we analyzed statistically. Statements about the number of neurons showing specific effects are made with reference to these task-related neurons. The number of neurons is similar to those reported in previous studies on primate amygdala; we performed no formal sample size estimation. Thus, experiments were replicated across both animals and across recorded neurons. Animals were not assigned to different groups; accordingly, randomization and blinding were not performed. No animals or recorded neurons were excluded.

Following completion of data collection, the animals received an overdose of pentobarbital sodium (90 mg/kg iv) and were perfused with 4% paraformaldehyde in 0.1 M phosphate buffer through the left ventricle of the heart. We reconstructed recording positions from 50-μm-thick, stereotaxically oriented coronal brain sections stained with cresyl violet based on electrolytic lesions (15–20 μA, 20–60 s, made in one animal) and lesions by cannulas placed to demarcate recording areas, recording coordinates for individual neurons noted during experiments, and in reference to other brain structures with known electrophysiological signatures recorded during experiments (internal and external globus pallidus, substantia innominata). We assigned recorded neurons to amygdala subnuclei with reference to a stereotaxic atlas (Paxinos et al., 2000) at different anterior-posterior positions (figures show neuron locations collapsed over anterior-posterior levels). We recorded 66 neurons from the lateral amygdala, 86 neurons from the basolateral amygdala, 23 neurons from the basomedial (also termed accessory basal) amygdala and 30 neurons from the centromedial amygdala (Table S6). The histological reconstructions validated also the previously radiographically assessed anatomical position of the amygdala as done in earlier reports (Grabenhorst et al., 2012).

#### Observational learning task

Two monkeys performed an observational learning decision-making task (probabilistic reversal learning) under computer control (Figure 1A). The animals sat in primate chairs (Crist Instruments) and faced each other over a horizontally mounted touch screen (EloTouch 1522L 15’; Tyco). The animals alternated trial-by-trial making choices between pairs of sequentially presented visual objects. The animals worked on separate object pairs; we switched object pairs between animals halfway through an experimental session. To maximize reward, the animals were required to learn and track the (uncued) reward probabilities associated with the different objects. One object within a pair was associated with a reward probability of 0.85, whereas the other object was associated with a reward probability of 0.15. Reward probabilities reversed between objects after blocks of typically 25 – 35 trials per animal. The specific reward probabilities were chosen based on pre-testing to ensure that the animals maintained high motivation during the task while at the same time providing sufficient variation in choices. A computer-controlled solenoid valve delivered juice reward from a spout in front of the animal’s mouth. On each completed trial, the acting animal received one of two outcomes: on ‘rewarded’ trials, a liquid reward of 0.8 mL was delivered whereas on ‘non-rewarded’ trials, a small reward of 0.05 mL was delivered. The observer animal did not receive any reward. We found that a small reward instead of non-reward on ‘unrewarded’ trials ensured that the animal maintained high motivation on this demanding task, in which each animal was rewarded only every second trial. Reward delivery of both large and small reward was accompanied by a sound to mask solenoid clicks.

The outline of a recording session is shown in Figure 1B and a full trial-by-trial record for one session for both animals is shown in Figure 2A. Each session consisted of four main periods. At session start, the animals took turns trial-by-trial to choose between two novel visual cues (‘objects’), with each animal choosing from its own object pair (two object pairs, four objects in total per session). Depending on the animals’ learning performance, typically after 25-35 trials, we reversed the reward probabilities between the two objects in each session, requiring the animals to adapt their choices to maximize reward. Following another period of 25-35 trials, we switched the object sets between animals (‘object switch’), crucially without altering the object-reward probabilities. This design allowed the animals to observe each other’s choice before object switch to learn the current reward value of each object, and subsequently use this knowledge for their own choices once objects switched between animals. After object switch, we performed another reward-probability reversal. Thus, an average recording of one neuron would consist of about 200 choice trials. (The raster plots in the figures show only a subset of these trials while corresponding peri-event time histograms were calculated based on all recorded trials for a given neuron.)

Each trial started when the background color on the touch screen changed from black to gray. To initiate the trial, both recorded monkey and partner monkey were required to place their hand on an immobile, touch-sensitive key (each animal had its own touch key). Presentation of the gray background was followed by presentation of an ocular fixation spot (1.3° visual angle). On each trial, the recorded animal was then required to fixate this spot within 4° for 500 ms. Following 500 ms of central fixation, a first choice cue (‘object’) appeared centrally for 350 ms and was followed, after cue offset, by a 350 ms inter-stimulus interval, which was then followed by a second choice cue shown for 350 ms and another 350 ms inter-stimulus interval. (A few initial recording sessions used durations of 500 ms.) Following sequential presentation of these individual choice objects, the two objects reappeared simultaneously on the left and right side of the monitor (determined pseudorandomly). After 100 ms, the fixation spot disappeared and two blue rectangles appeared below the choice objects at the margin of the monitor, close to the position of the touch-sensitive key on the side of the acting animal that was required to choose on the current trial. The recorded animal was no longer required to fixate once the fixation spot had disappeared. The acting animal was required to release the touch key and touch one of the object-associated blue rectangles within 1.5 s to make its choice. Once the animal’s choice was registered, the unchosen object disappeared and after a delay of 500 ms, the chosen object also disappeared and a liquid reward was given to the acting animal. Reward delivery was followed by a trial-end period of 1,000 – 2,000 ms which ended with extinction of the gray background. The next trial started after an inter-trial interval of 2,000 – 4,000 ms (drawn from a uniform random distribution). The roles of acting and non-observing animal reversed after every correct trial. Assignment of visual objects to first or second presentation period and to left or right choice target position on each trial was randomized.

Possible errors included failure to make contact with the touch-sensitive key before the trial (both animals), key release before the go signal (both animals), failure to touch a choice target (acting animal), failure to fixate the central fixation spot at trial start (recorded animal) or fixation break in the period between initial fixation and disappearance of fixation spot (recorded animal). Errors led to a brief time out (3,000 ms) with a black background and then trial repetition. Task performance was typically interrupted after three consecutive errors.

Stimuli and behavior were controlled using custom MATLAB code (The Mathworks) and Psychophysics toolbox (version 3.0.8). The laboratory was interfaced with data acquisition boards (NI 6225; National Instruments) installed on a PC running Microsoft Windows 7.

#### Non-social control task

We tested some amygdala neurons in a non-social control task to determine whether neuronal value coding in the observational-learning task was specific to a social situation with decision-making requirements. The control task involved presentation of pre-trained conditioned stimuli that predicted liquid reward for the recorded monkey with different probabilities. This separate task was performed without the social partner being present in the room; it thus constituted an entirely non-social situation. Note that the recorded monkey’s own trials during the main task already constituted a non-social control for neuronal decision activity with the partner being present. Each trial started with presentation of a fixation spot. The animal was required to fixate within 4° for 500 ms and throughout the trial until reward delivery. The fixation spot was followed by presentation of a visual conditioned stimulus (drawn from a set of four to six stimuli) in the center of the screen for 500 ms; stimuli were distinct from but similar to the ones used in the main observational learning task. Each stimulus predicted forthcoming reward with a specific probability between 0.15 and 0.85. This stimulus period was used for neuronal data analysis by regressing impulse activity in this period on cued reward probability. Stimulus presentation was followed by an inter-stimulus interval of 500 ms, which was followed by reward delivery. In some cases, we included an additional 500 ms reward magnitude cue with subsequent 500 ms inter-stimulus interval before reward delivery. Separate choice trials using the stimuli from this control task verified that the animals could use the information provided by these stimuli to make meaningful, reward-maximizing choices (preferring higher over lower reward probabilities and higher over lower reward magnitudes).

### Quantification and Statistical Analysis

#### Behavioral data analysis

To assess the animals’ speed of learning, we calculated the number of trials that the animal required to choose the object with the current high reward probability, separately for session start (individual learning) and after object switch between animals (observational learning) (Subiaul et al., 2004). We then compared trial numbers for individual learning and observational learning across recording sessions (two-sided paired t test, Figure 2B).

To test whether the animals’ choice for the high-probability object post-switch depended on partner’s preceding choices (Figure S1A), we proceeded as follows. We regressed the recorded monkeys’ choice probability for the high-probability object within the first three trials following object switch in each session on two variables: the first was a dummy variable indicating whether the partner monkey chose the high-probability object on the last trial before switch (this was the critical variable that captured the potential observational learning effect: the recorded monkey might be more likely to choose the high-probability object if he observed the partner choose this object immediately before object switch); the second regressor was a control dummy variable indicating whether the recorded monkey chose the high-probability object on the last trial before switch (as this variable referred to a different object than the one assessed in the post-choice period, it should be unrelated to the recorded monkey’s post-switch choice).

#### Reinforcement learning model

To describe the animals’ behavior in the observational learning task, and to derive trial-by-trial measures of subjective object values for neuronal analysis, we fitted reinforcement learning models to the animals’ choices. We fitted separate models to each animal’s own choice-reward records. (Note that our study did not aim to test how an animal’s own choices and rewards were integrated on a trial-by-trial basis with the partner’s observed choices and rewards; such a test would require that the animals take turns choosing between the same object pair trial-by-trial, as opposed to working on separate object pairs as done here.)

The best-fitting model (‘Reversal RL’, see Table S1) accounted for the reversal-learning nature of the task by updating both the value of the chosen and unchosen option on each trial, as typical for reward-reversal learning tasks. Object values in this model were updated as follows (Equation 1):

$$V_{A}^{t+1}=V_{A}^{t}+\;\alpha \left(R^{t}-V_{A}^{t}\right)$$

$$V_{B}^{t+1}=V_{B}^{t}+\;\alpha \left(-R^{t}-V_{B}^{t}\right)$$

with $V_{A}^{t}$ as the expected value of object A on trial t, $R^{t}$ as reward (coded as 0 or 1 for small and large reward, respectively), $R^{t}-V_{A}^{t}$ as prediction error between reward $R^{t}$ and expected value $V_{A}^{t}$ on trial t, α as free-parameter learning rate and $V_{A}^{t+1}$ as the updated expected object value for the next trial, and corresponding variables for the alternative object B. The prediction error for object B,$\;-R^{t}-V_{B}^{t}$, involved updating the value for object B in the opposite direction as for object A. This model is a variant of standard reinforcement learning as it updates additionally the value of the unchosen option. The object choice on each trial was determined by the softmax rule (Sutton and Barto, 1998) (Equation 2):

$$P\left(A\right)=\;\frac{1}{\left(1+\exp\left(-\beta \left(V_{A}^{t}-V_{B}^{t}\right)\right)\right)}\;$$

with P(A) as choice probability for object A and β as the free-parameter inverse temperature, which reflects the degree of stochasticity in the animal’s choices.

We estimated the model’s free parameters by fitting the model to the trial-by-trial record of choices and rewards within each session, separately for each session and separately for the two animals. Model fitting was performed using a maximum likelihood procedure with the Nelder–Mead search algorithm (implemented by the MATLAB function ‘fminsearch’).

We compared several alternative reinforcement learning models with the results of the model comparison shown in Table S1. The additional models tested include: (1) a reinforcement learning model formulated as above but without updating the value of the unchosen option (‘Basic RL’ in Table S1), (2) a reinforcement learning model with an adaptive learning rate, whereby the learning rate on each trial was modified based on the unsigned trial-specific reward prediction error (‘Reversal RL, adaptive rate’), (3) variants of the reinforcement learning models just described with different learning rates for the chosen and unchosen option (‘Reversal RL, 2 learning rates’). Model comparison using Akaike Information Criterion (AIC), Bayesian Information Criterion (BIC) and Pseudo R^2^ identified the reversal-learning variant as the best-fitting model.

#### Eye data analysis

We monitored the recorded animal’s eye position using an infrared eye tracking system at 125 Hz (ETL200; ISCAN) placed next to the touchscreen. Before each recording session, we calibrated the eye tracker during a fixation task with a moving fixation spot that the animals had to follow. During recordings, accuracy of calibration of the eye tracker was regularly checked and if necessary recalibrated. The monkey’s head was slightly tilted forward (∼10°) for a better view of the touchscreen. We assessed eye position in a plane in front of the monkey’s eyes, followed by a transformation to the horizontal touchscreen plane (Báez-Mendoza et al., 2013). We then determined whether and when a fixation occurred. We defined a fixation when eye velocity was below 25% of its statistical standard deviation for more than 60 ms. For analysis of fixations in specific task-related time windows, we excluded fixations that occurred within the first 100 ms of stimulus onset to remove anticipatory fixations. We selected fixations that met the above criteria and that occurred on specific trial types, e.g., other’s trial, left chosen. To create frequency maps of eye fixations, a histogram matrix (50 × 50 cm) with the possible eye positions was convolved with a Gaussian function (σ = 1.5). Matrices were then converted into percentage units. To obtain the maps shown in Figure 2D and Figure S1E, we subtracted the fixation matrix of left chosen trials from the matrix for right chosen trials to obtain a matrix of differential left-right eye fixations (thus, a positive difference means that a higher percentage of fixations occurred for a given location on left-chosen trials compared to right-chosen trials). We rescaled the color map so that zero difference was shown in white color. For statistical comparisons, we defined regions of interest (ROIs) around left and right choice objects, which were given by the position of the object on the screen; we defined these positions for each monkey based on plots during the period where only the chosen object was shown and the animal looked at that object. Statistical comparisons were performed for fixation densities across all coordinates within the ROIs. We performed a ranksum test to compare fixation frequency differences between the left and right object regions of interest. For analysis of gaze patterns during choice target presentation and before confirmation of choice, we focused on fixations that lasted a minimum of 500 ms, which in many cases selected the final fixation before key release. Results remained significant for a shorter analysis period between choice cue onset and before release of the touch key, i.e., before the animal initiated a movement to execute choice. For analyses shown in Figure S5, we were interested in the recorded monkey’s dynamic gaze patterns during observation of the partner’s choices, and therefore used a shorter minimum fixation criterion of 300 ms.

#### Neuronal data analysis

We counted neuronal impulses for each neuron on correct trials in fixed time windows relative to different task events focusing on the following non-overlapping task epochs: 500 ms after fixation spot before cues (Fixation), 350 ms after onset of first cue (i.e., first choice object), 350 ms after offset of first cue, 350 ms after onset of second cue, 350 ms after offset of second cue, 500 ms after onset of choice targets. We did not observe systematic differences in activity patterns between animals in preliminary analyses; therefore, we pooled data from both animals for subsequent analyses.

Our analysis strategy was as follows. We used fixed-window and sliding-window linear regression analyses to identify neuronal responses related to specific variables. For fixed-window analyses, we first identified task-related object-evoked responses by comparing activity during object presentation (first and second cue period) to a baseline control period (before appearance of fixation spot) using the Wilcoxon test (p < 0.005, Bonferroni-corrected for multiple comparisons). A neuronal response was classified as task-related if it was significantly different to activity in the control period (the pre-fixation period on each trial of the main social task). We used a multiple linear regression model to test for neuronal activities related to specific task variables while including other relevant variables as covariates. We also used sliding-window multiple regression analyses with a 200-ms window that we moved in steps of 25 ms across each trial (without pre-selecting task-related responses). Sliding-window analyses tested for dynamic coding of different task-related variables over time within trials and also confirmed that our results did not depend on the pre-selection of task-related responses or definition of fixed analysis windows. To determine significance of sliding-window regression coefficients, we used a bootstrap approach as follows. For each neuron, we performed the sliding-window regression 1,000 times using trial-shuffled data and determined a false positive rate by counting the number of consecutive sliding-windows in which a regression was significant with p < 0.05. We found that less than five percent of neurons with trial-shuffled data showed more than seven consecutive significant analysis windows. Accordingly, we classified a sliding-window analysis as significant if a neuron showed a significant (p < 0.05) effect for more than seven consecutive windows. Statistical significance of regression coefficients was determined using t test; all tests performed were two-sided. Additional population decoding, described below, examined independence of our findings from pre-selection of task-related responses and served to assess information about specific task variables contained in the neuronal population.

We performed our regression analysis in the framework of the general linear model (GLM). Neuronal responses were tested with the following regression models:

GLM 1 (Equation 3): this GLM was the main model for identification of object-value coding responses. It served the following purposes: First, the GLM served to identify neurons whose object-evoked responses encoded value across animals. Second, the GLM served to derive value βs for the histogram shown in Figure 3C.

$$y=\;\beta_{0}+\;\beta_{1}\left(Object\;value\right)\;+\;\beta_{2\;}\left(Self-other\right)+\;\beta_{3}\;Choice\;+\;\beta_{4\;}\left(Object\;sequence\right)\;+\;\varepsilon \;$$

with y as the neuronal activity in response to a specific object during the 350 ms period in which the object was shown on each trial (measured over the whole experimental session, including pre-switch and post-switch periods), ‘Object value’ as the trial-specific subjective value of that object as derived from a reinforcement learning model (Equation 1) fitted to the choices of the animal that was currently working on that object, ‘Self-other’ as a dummy variable (coded as 1 or 0) indicating whether the current trial was for the recorded monkey (self) or the partner (other), ‘Choice’ as the current-trial object choice (coded as 1 or 0 if the object was chosen or not chosen, respectively), ‘Object sequence ‘’as a dummy variable for the current-trial object sequence (coded as 1 or 0 depending on whether the object was shown first or second on the current trial, respectively), and ε as error. Note that per neuron, we fitted the GLM four times on distinct object-evoked responses as we tested four objects per neuron; we thus tested 820 object responses (4 objects × 205 neurons). Note that the datasets for each object within each neuron were independent from each other because we showed objects sequentially and thus could measure neuronal responses evoked by specific objects.

GLM 2 (Equation 4): this GLM served as a test for coding of object values that were derived purely from observation, by testing for object-value coding before objects switched between animals, and thus before the recorded monkey experienced own reward from the partner’s objects.

$$y=\;\beta_{0}+\;\beta_{1}\;\left(Object\;value\right)\;+\;\beta_{2\;}\;Choice\;+\;\beta_{3\;}\left(Object\;sequence\right)+\;\varepsilon \;$$

with y as the neuronal activity in response to a specific object before objects switched between animals, and all other variables as defined above. This GLM 2 identified 113 value neurons on Self trials and 46 value neurons on Other trials. These latter neurons thus unambiguously encoded object values derived from observation, before the recorded monkey chose any of the partner’s objects.

GLM 3 (Equation 5): this GLM served the following purposes: First, to test how many neurons had activity related to object values separately on Self trials and on Other trials. Second, to determine how many neurons had activity related to object values for both Self and Other trials when relationships to object value were assessed with separate GLMs for Self and Other trials (thus, lowering statistical power, but showing unambiguous value coding on Self and Other trials). Third, the GLM served to derive value βs for the analyses shown in Figure 3H, I.

$$y=\;\beta_{0}+\;\beta_{1}\;\left(Object\;value\right)\;+\;\;\beta_{2}\;Choice\;+\;\beta_{3\;}\left(Object\;sequence\right)\;+\;\varepsilon \;$$

with y as the neuronal activity in response to a specific object on Self trials or Other trials across the whole experimental session. This GLM 3 identified 232 value responses (130 neurons) on Self trials and 101 value responses (72 neurons) on Other trials. Of these neurons, 53 neurons showed significant value coding on both Self trials and Other trials (when assessed with separate GLMs). This result, together with the significant relationship between value coefficients for Self and Other (Figure 3H), supports the conclusion that many amygdala neurons encoded object values irrespective of whether value derived from individual learning or observational learning.

GLM 4 (Equation 6) this GLM served to test whether overall model fit of our main value-coding model GLM1 was improved by inclusion of separate interaction terms that modeled value-coding specifically for self-trials and specifically for other trials. We used partial F-tests to test for significant (p < 0.05) improvements in model fit by inclusion of self-other specific value regressors, compared to GLM 1:

$$y=\;\beta_{0}+\;\;\beta_{1\;}\left(Self-Other\right)\;+\;\beta_{2}\;Choice\;+\;\beta_{3\;}\left(Object\;sequence\right)+\;\beta_{4}\;\left(Object\;value\;\times Self\right)+\;\beta_{5}\;\left(Object\;value\;\times Other\right)+\;\varepsilon \;$$

with y as the neuronal activity in response to a specific object across the whole session, ‘Object value × Self’ as an interaction term between object value and Self - Other and ‘Object value × Other’ as an interaction term between object value and Self - Other trials (the two interaction terms thus tested for object value coding specifically on self or other trials). This GLM 4 showed that of 221 value-coding responses identified with GLM 1, the majority (155 responses, 70%) were not significantly improved by modeling Self/Other-specific value coding. These results support our conclusion that value coding occurred mostly irrespective of the self versus other distinction.

GLM 5 (Equation 7): this GLM was the main model for identifying neurons with significant choice-coding and significant coding of first-object value and second-object value. It served the following purposes. First, the GLM served to identify neurons with activity related to choices and dynamic value comparisons, while controlling for other task-related variables. Second, the GLM served to derive coefficients of partial determination (partial R^2^) for Figure 4E, Figure S3E and Figure S4B. Third, the GLM served to derive regression coefficients (βs) for the analyses in Figure S5. Two GLMs were fitted separately to data on recorded monkey’s trials and data on partner’s trials. The GLM was calculated as a sliding-window multiple regression (except for analyses shown in Figure S5, for which we used fixed-window analysis to derive neuronal βs).

$$y=\;\beta_{0}+\;\beta_{1\;}\left(Object1\;first-Object2\;first\right)+\;\beta_{2\;}\left(Object3\;first-Object4\;first\right)+\;\beta_{3\;}\left(Object1\;chosen-Object2\;chosen\right)+\;\beta_{4\;}\left(Object3\;chosen-Object4\;chosen\right)+\;\beta_{5}\;\left(First\;object\;chosen\right)\;+\;\beta_{6\;}\left(First\;object\;value\right)+\;\beta_{7}\;\left(Second\;object\;value\right)+\;\beta_{8}\;\left(Chosen\;object\;value\right)+\;\varepsilon$$

with y as neuronal activity in 200 ms windows that were moved in 25 ms steps across the trial, starting 500 ms before onset of first cue and ending 350 ms after onset of choice targets, ‘Object1 first – Object2 first’ as indicator variable for whether object 1 or object 2 was shown as first cue on a given trial, ‘Object3 first – Object4 first’ as indicator variable for whether object 3 or object 4 was shown as first cue on a given trial, ‘Object1 chosen – Object2′ chosen as indicator variable for whether object 1 or object 2 was chosen on a given trial, ‘Object3 chosen – Object4’ chosen as indicator variable for whether object 3 or object 4 was chosen on a given trial, ‘First object chosen’ as indicator variable for whether the first or second object was chosen on a given trial, ‘First object value’ as object value of the first shown object, ‘Second object value’ as object value of the second shown object, ‘Chosen object value’ as the object value of the chosen object. Objects 1 to 4 were defined according to the pairing of objects for recorded monkey and partner as follows. Objects 1 and 2 were the objects from which the recorded monkey chose at session start; following object switch, the partner chose between these objects. Objects 3 and 4 were the objects from which the partner monkey chose at session start; following object switch, the recorded monkey chose between these objects.

We also used this GLM 5 to examine whether object-value neurons identified in GLM 1 (Equation 3) were distinct from neurons that encoded values for decision-making (in an order-based reference frame of first-versus-second object) identified in GLM5 (Equation 7). Among 127 neurons classified as object-value coding with GLM 1, 45 neurons (35%) were also classified as coding values for decision-making on partner’s trials (‘Second object value’ regressor in Equation 7). By contrast, 82 neurons encoded object value but were insignificant for the second-value regressor on partner’s trials and 30 neurons were significant for the second-value regressor on partner’s trials without showing object-value coding. For the recorded monkey’s trials, 53 object-value neurons (43%) showed a significant second-value regressor, 74 neurons encoded object value but were insignificant for the second-value regressor and 28 neurons were significant for the second-value regressor on recorded monkey’s trials without showing object value. Thus, some neurons were classified as coding both object value and value for decision-making but substantial numbers of neurons also showed distinct coding of either object value or order-based value for decision-making.

GLM 6 (Equation 8): this GLM served the following purposes. First, the GLM served to identify neurons with activity that distinguished self versus other trials, while controlling for other task-related variables. Second, the GLM served to derive self-other βs for the graph shown in Figure 6C. Third, the GLM served to calculate coding latencies for Figure 7F. Fourth, the GLM served to derive coefficients of partial determination (partial R^2^) for Figure 7G. One GLM was fitted across both recorded monkey’s and partner’s trials. The GLM was calculated as a sliding-window multiple regression.

$$y=\;\beta_{0}+\;\beta_{1\;}\left(Object1\;first-Object2\;first\right)\;+\;\beta_{2\;}\left(Object3\;first-Object4\;first\right)\;+\;\beta_{3\;}\left(Object1\;chosen-Object2\;chosen\right)+\beta_{4\;}\left(Object3\;chosen-Object4\;chosen\right)\;+\;\beta_{5}\;\left(First\;object\;chosen\right)\;+\;\beta_{6\;}\left(First\;object\;value\right)+\;\beta_{7}\;\left(Second\;object\;value\right)+\beta_{8}\;\left(Chosen\;object\;value\right)+\;\beta_{9}\;\left(Self-Other\right)+\;\varepsilon$$

with ‘Self – Other’ as an indicator variable for whether the current trial was the recorded monkey’s or the partner’s trial, and all other variables as defined above.

GLM 7 (Equation 9): this GLM served to derive value βs for the analyses and plots shown in Figure 4C and Figure S3C and for selecting responses for the plots shown in Figure 4B (N = 37 responses) and Figure S3B (N = 107 responses). Separate GLMs were fitted for recorded monkey’s and partner’s trials. We used this model to estimate the valuation component of neuronal decision-related activities, as was done in previous studies (Strait et al., 2014), for the purposes of performing the analysis shown in Figure 4C and S3C, and for visualizing the value-comparison effect in population activity shown in Figure 4B and Figure S3B. The formal identification of neurons with significant dynamic value-coding was performed with GLM 5.

$$y=\;\beta_{0}+\;\beta_{1\;}\left(Object\;value\right)+\;\varepsilon \;$$

with y as firing rate during presentation of first cue or second cue (fitted in separate GLMs) and ‘Object value’ as object value of the first or second object (fitted in separate GLMs).

GLM 8 (Equation 10): this GLM served the following purposes. First, the GLM served to identify neurons with activity related to recorded monkey’s or partner’s left-right actions and object-specific spatial left-right cue positions. Second, the GLM served to derive coefficients of partial determination (partial R^2^) for Figure S6B and E. Two GLM were fitted separately for recorded monkey’s and partner’s trials. The GLM was calculated as a sliding-window multiple regression.

$$y=\beta_{0}+\;\beta_{1\;}\left(Object1\;chosen-Object2\;chosen\right)\;+\;\beta_{2\;}\left(Object3\;chosen-Object4\;chosen\right)\;+\;\beta_{3\;}\left(First\;object\;chosen\right)+\;\beta_{4\;}\left(First\;object\;value\right)+\;\beta_{5}\;\left(Second\;object\;value\right)\;+\beta_{6}\;\left(Chosen\;object\;value\right)+\;\beta_{7}\;\left(Object1\;left-Object2\;left\right)+\;\beta_{8}\;\left(Object3\;left-Object4\;left\right)\;+\;\beta_{9}\;\left(Left\;chosen\right)+\;\varepsilon \;$$

with y as firing rate in 200 ms windows that were moved in 25 ms steps across the trial, starting 350 ms before onset of choice targets and ending 750 ms after onset of choice targets, ‘Object1 left – Object2 left’ as indicator variable for whether object 1 or object 2 was shown as left or right choice target, ‘Object3 left – Object4’ left as indicator variable for whether object 3 or object 4 was shown as left or right choice target, ‘Left chosen’ as indicator variable for whether the left or right target was chosen.

#### Change point analysis

We performed a change point analysis to test the correspondence between activity of value-coding neurons and the animals’ choices following unannounced probability reversals, using methods used in previous studies (Paton et al., 2006). The test identifies change points based on slope changes in the cumulative record of choices and neuronal responses. We constructed cumulative records of neuronal activity around reversal trials based on seven-trial smoothed neuronal impulse rates measured during object presentation. We constructed corresponding cumulative choice records around reversal points. We included data from both recorded animal and partner. We excluded sessions in which no change point was identified. Following previous studies (Paton et al., 2006), if multiple change points were identified, we picked the one closest to the reversal point. A change point was identified using t test with a critical value of p = 1.0 × 10^−6^. We show in Figure 3E the results from 182 value-coding responses that met these criteria (R = 0.264, p = 0.0003). The result was significant individually within each monkey (monkey A: R = 0.288, p = 0.002; monkey B: R = 0.242, p = 0.04) and similar results were obtained across all responses, without pre-selection for value coding (across animals: R = 0.166, p = 5.5 × 10^−5^; monkey A: R = 0.233, p = 4.2 × 10^−5^; monkey B: R = 0.119, p = 0.048).

#### Normalization of population activity

To normalize activity from different amygdala neurons, we subtracted from the impulse rate in a given task period the mean impulse rate of the pre-fixation control period and divided by the standard deviation of the control period (z-score normalization). We also distinguished neurons that showed positive relationships or negative relationships with a given variable, based on the sign of the regression coefficient, and sign-corrected responses with a negative relationship. Normalized data were used for Figure 3D, Figure 4B, D, Figure S2A, C, D, Figure S3B, D, Figure 6B, and all decoding analyses.

#### Normalization of regression coefficients

Standardized regression coefficients were defined as x_i_(s_i_/s_y_), *x*_*i*_ being the raw slope coefficient for regressor *i*, and *s*_*i*_ and *s*_*y*_ the standard deviations of independent variable *i* and the dependent variable, respectively. Standardized regression coefficients were used for Figure 3C, 3H, 3I, Figure 4C, Figure 6C, Figure S2C, Figure S3C and Figure S4D. Specifically, to create the scatterplots shown in Figure 4C and Figure S3C, we performed a linear regression of impulse rates during presentation of the first object or second object on that object’s current value (Equation 9), derived from the animal-specific reinforcement-learning model. These regressions were performed separately for recorded monkey and partner monkey. The scatterplots show data from all 205 recorded amygdala neurons (one data point lies outside the plotted data range).

#### Population decoding

We used a support-vector-machine (SVM) classifier to quantify information about task-related variables contained in neuronal population activity in defined task periods, following previous neurophysiological studies (Grabenhorst et al., 2016, Tsutsui et al., 2016). The SVM classifier was trained to find a linear hyperplane that best separated patterns of neuronal population activity defined by a given grouping variable (e.g., high versus low value, choice for object A versus object B, self-trial versus other-trial, self-choice versus other-choice). Additional nearest-neighbor (NN) classification was also used which assigned each trial to the group of its nearest single-trial neighbor in a space defined by the distribution of impulse rates for different levels of the grouping variable using the Euclidean distance. Both SVM and NN classification are biologically plausible as downstream neurons could perform similar classification by comparing inputs on a given trial with stored synaptic-weight vectors. Both classifiers performed qualitatively very similar but SVM decoding was typically more accurate.

To prepare data for decoding, we aggregated z-normalized trial-by-trial impulse rates of independently recorded amygdala neurons from specific task periods into pseudo-populations. We used all recorded neurons that met inclusion criteria for a minimum trial number, without pre-selecting for coding a specific variable. Depending on the variable used for decoding, we only included neurons in the decoding analyses that had a minimum number of either 5 or 10 trials per group for which decoding was performed; we confirmed that results were robust to changes in this minimum trial number. We created two *n* by *m* matrices with *n* columns determined by the number of neurons and *m* rows determined by the number of trials. We defined two matrices, one for each group for which decoding was performed, using the following different groupings. For object-value decoding, we defined separate groups for low and high object value, determined for each neuron by calculating value terciles. (We obtained very similar results by repeating the decoding analyses based on median-split.) For choice decoding, we defined two separate groups depending on the object choice on each trial (A or B, given by the set of two objects from which the animal was currently choosing). For self-other decoding, we defined two separate groups depending on whether it was the recorded monkey’s (self) or partner’s (other) trial. For choice decoding shown in Figure 7H, we wished to test the neuronal discriminability of self-choice versus other-choice; we therefore grouped trials according to whether recorded monkey or partner chose a given object. Accordingly, each cell in a matrix contained the impulse rate from a single neuron on a single trial measured for a given group. Because neurons were not simultaneously recorded, we randomly matched up trials from different neurons for the same group and then repeated the decoding analysis with different random trial matching (within-group trial matching) 150 times. We found this number of repetitions produced very stable classification results and confirmed robustness with respect to changes in this number. (We note that this overall approach likely provides a lower bound for decoding performance as it ignores potential contributions from cross-correlations between neurons; investigation of cross-correlations would require data from simultaneously recorded neurons.)

We quantified decoding accuracy as the percentage of correctly classified trials, averaged over all decoding analyses for different random within-group trial matchings. We used a leave-one-out cross-validation procedure: a classifier was trained to learn the mapping from impulse rates to groups on all trials except one test trial; this remaining trial was then used for testing the classifier and the procedure repeated until all trials had been tested. We obtained very similar results when splitting data into 80% training trials and 20% test trials. We implemented SVM decoding in MATLAB (Version R2013b, Mathworks, Natick, MA) using the ‘svmtrain’ and ‘svmclassify’ functions with a linear kernel and the default sequential minimal optimization method for finding the separating hyperplane. The NN decoding was implemented in MATLAB with custom code.

To investigate how decoding accuracy depended on the number of neurons in the decoding sample (Figure 3J, Figure 6D), we randomly selected a given number of neurons at each step (without replacement) and then determined the percentage correct classification. For each step (i.e., each possible population size) this procedure was repeated 100 times. We also performed decoding for randomly shuffled data (shuffled group assignment without replacement) with 5,000 iterations to test whether decoding on real data differed significantly from chance. Statistical significance was determined by comparing vectors of percentage correct decoding accuracy between real data and randomly shuffled data using the rank-sum test.

For the analyses shown in Figures 3I, K, Figure 4G, I, and Figure S5C, D, we performed decoding repeatedly over 5,000 iterations based on small subsets (N = 20) of randomly sampled neurons drawn without replacement (within each iteration) from all neurons that met minimum criteria for classification. For each iteration, we noted the percentage-correct accuracy as well as the identity of the neurons included in the sample. This approach allowed us to then relate the decoding accuracy resulting from a given subset of 20 neurons to the average value slope of these individual neurons (Figure 3I), the average behavioral performance of the recorded monkey during the sessions in which these neurons were recorded (Figure 3K, Figure 4G), the fraction of basomedial neurons in the sample (Figure 4I), or mean fixation durations (Figure S5C, D).

For Figure 5, we adapted a nearest-neighbor classifier to examine coding across amygdala nuclei. We computed Euclidean distances between single-trial activity vectors and mean activity vectors for different value levels or choices. Such decoding could be neurally implemented by comparing current-trial activity patterns to synaptic-weight vectors based on past trials. We focused on the task period before objects switched between animals, as this was the most relevant period for observational learning. We preselected the 20 neurons with highest value-coding and separately the 20 neurons with highest choice coding in each nucleus, based on regression coefficients. We then proceeded as for the decoding analyses described above, except that decoding was not based on Euclidean distances between single-trial vectors but on Euclidean distances between a single-trial test vector and the mean activity vectors for the two alternative groups, calculated from all trials except the test trial. For Self-to-Other cross-decoding, we used single-trial test vectors from the recorded monkey’s trials and computed the Euclidean distances to mean activity vectors calculated from the partner’s trials. Figure 5A illustrates this approach: a single-trial test point (in the activity space of two example neurons) was compared to mean activity vectors for recorded monkey (upper panel) and partner (lower panel). Figure 5E and F show the correlations between the mean activity vectors corresponding to different decoding groups (i.e different value levels, or different choices) on recorded monkey’s trials (r_Self,Self_), different decoding groups on recorded monkey’s trials (r_Other,Other_), and same decoding groups on recorded monkey’s and partner’s trials (r_Self,Other_). A higher coefficient r_Self,Other_ compared to r_Self,Self_ and r_Self,Other_ would show that population vectors for the same values across recorded monkey’s and partner’s trials were more similar than vectors for different values within each animal, indicative of shared neuronal coding.

#### Biophysical neuronal network model of decision-making

We adapted an established network model of decision-making that we extended to the architecture shown in Figure 7A. The network contains two decision modules: the ‘self’ module computes the recorded monkey’s own choices and the ‘other’ module simulates the social partner’s choices. Each decision-making module is implemented as an attractor neural network (ANN), which is a widely studied model of evidence accumulation that relies on reverberant activity of competing neural populations (or pools) and mutual inhibition mediated by slow NMDA channel opening dynamics. The decision of each ANN depends on the activity of the competing decision populations. More specifically, each module is thus composed of two reverberating populations of excitatory object-specific decision-making neurons. The competition between the two alternative choices is implemented through mutual GABAergic inhibitory connections between both excitatory pools. The operation of each of these populations is captured by the Dynamic Mean Field (DMF) equations (Wong and Wang, 2006). The DMF describes consistently the time evolution of the ensemble activity of different neural populations consisting of biophysical realistic spiking neurons coupled through excitatory (AMPA and NMDA) and inhibitory (GABA-A) synaptic receptor types. In the DMF approach, each population firing rate depends on the input currents into that population, whereas the input currents depend on the firing rates. Consequently, the population firing rate can be determined self-consistently by a reduced system of coupled non-linear differential equations expressing the population firing rates and the respective input currents. In brief, the mean field approach considers the diffusion approximation according to which sums of synaptic gating variables are replaced by a DC component and a Gaussian fluctuation term. Moreover, the first passage equation for calculating the firing rate is approximated by a simple sigmoidal input–output function (Wong and Wang, 2006). Since the synaptic gating variable of NMDA receptors has a much longer decay time constant (100 ms) than the AMPA receptors, the dynamics of the NMDA gating variable dominates the time evolution of the system, while the AMPA synaptic variable instantaneously reaches its steady state. Hence, one can neglect contributions by the AMPA receptors to the local recurrent excitation. The decision-making module dynamics can be simply described by the following set of coupled non-linear stochastic differential equations:

$$x_{1}\left(t\right)=J_{11}S_{1}\left(t\right)-J_{12}S_{2}\left(t\right)+I_{0}+I_{1}+I_{noise,1}\left(t\right)$$

$$x_{2}\left(t\right)=J_{22}S_{2}\left(t\right)-J_{21}S_{1}\left(t\right)+I_{0}+I_{2}+I_{noise,2}\left(t\right)$$

$$\frac{dS_{i}\left(t\right)}{dt}=-\frac{S_{i}\left(t\right)}{\tau_{S}}+\left(1-S_{i}\left(t\right)\right)\gamma H_{i}\left(t\right)$$

$$H_{i}\left(t\right)=\frac{ax_{i}\left(t\right)-b}{1-\text{exp}\left[-d\left(ax_{i}\left(t\right)-b\right)\right]}$$

$$I_{i}=J_{ext}\mu_{0}\left(1\mp D\right)$$

$$\tau_{n}\frac{dI_{noise,i}\left(t\right)}{dt}=-I_{noise,i}\left(t\right)+\omega \left(t\right)\sqrt{\tau_{n}\sigma_{noise}^{2}}$$

where $H_{i}\left(t\right)$ denotes the population firing rate of the excitatory population *i*. $S_{i}\left(t\right)$ denotes the average excitatory synaptic gating variable at the population *i*. The input currents to the excitatory population *i* is given by $I_{i}$. The different level of value evidence to the respective decision-making pools is regulated by the parameter D. In our case D was 0.01 for the difficult decision condition and 0.05 for the easy decision-making condition. The input parameters were $J_{ext}$ = 0.000183 nAHz^-1^ and $\mu_{0}$ = 30 Hz. $I_{0}$ encodes external social input stimulation for simulating the social effect. As shown in Figure 7E, $I_{0}$ was chosen before the bifurcation ($I_{0}$ = 0.38) for simulating the case without social input, and after the bifurcation ($I_{0}$ = 0.44) for simulating the case with social input. Parameter values for the neuronal input– output functions *H* are: *a* = 270 (VnC)^-1^, *b* = 108 Hz, and *d* = 0.154 s. The kinetic parameters are γ = 0.641, the NMDA latency $\tau_{S}$ = 100 ms and the noise latency $\tau_{n}$ = 10 ms. The reverberatory excitatory synaptic coupling was $J_{11}$ = $J_{22}$ = 0.6 nA and the inhibitory synaptic coupling was $J_{12}$ = $J_{21}$ = 0.3 nA. In the last equation ω(t) is uncorrelated standard Gaussian noise and the noise amplitude was $\sigma_{noise}^{2}$ = 0.02 nA. We used the Euler-Murayama method for integrating the stochastic system of coupled differential equations.
