# Supplementary material for: Primate Amygdala Neurons Simulate Decision Processes of Social Partners
Source: Cell. 2019 May 2;177(4):986–998.e15. doi: 10.1016/j.cell.2019.02.042 (PMC6506276; doi:10.1016/j.cell.2019.02.042)
Supplement: Document S1. Tables S1–S6 [file mmc1.pdf]

Cell, Volume *177*

Supplemental Information

Primate Amygdala Neurons Simulate Decision Processes of Social Partners

Fabian Grabenhorst, Raymundo Báez-Mendoza, Wilfried Genest, Gustavo Deco, and Wolfram Schultz

Supplemental Information

Primate amygdala neurons simulate decision processes of social partners

Fabian Grabenhorst, Raymundo Báez-Mendoza, Wilfried Genest, Gustavo Deco, and Wolfram Schultz

Table S1. Reinforcement learning models (related to Figure 2). Results from fitting reinforcement learning models to recorded monkeys' and partner monkey's choices (Eq. 1-2). Shown are median ± s.e.m. of % correctly modelled choices, Akaike information criterion (AIC), Bayesian information criterion (BIC), and Pseudo R^2.

	% correct modelled choices	AIC	BIC	Pseudo R^2
Recorded monkeys				
Basic RL	86.00 ± 0.5	64.20 ± 1.7	72.05 ± 1.8	0.54 ± 0.01
Reversal RL	88.78 ± 0.3	55.25 ± 1.3	65.67 ± 1.3	0.64 ± 0.01
Reversal RL, 2 learning rates	89.09 ± 0.3	56.13 ± 1.3	66.44 ± 1.4	0.62 ± 0.01
Reversal, RL, adaptive rate	89.00 ± 0.5	61.55 ± 1.3	71.85 ± 1.4	0.59 ± 0.01
Partner monkey				
Basic RL	84.16 ± 0.4	77.44 ± 1.9	84.98 ± 1.9	0.45 ± 0.01
Reversal RL	85.45 ± 0.5	67.60 ± 1.9	78.04 ± 2.0	0.53 ± 0.01
Reversal RL, 2 learning rates	85.86 ± 0.4	69.89 ± 1.9	80.31 ± 1.9	0.53 ± 0.01
Reversal, RL, adaptive rate	86.14 ± 0.6	68.61 ± 1.8	78.94 ± 1.9	0.52 ± 0.01

Table S2. Neuronal responses related to object value (related to Figure 3). Results of fitting GLM 1 (Eq. 3) to object-evoked neuronal responses. Numbers in parentheses are percentages.

	Total	Object value					
	Neurons	Neurons	Responses	1 object	2 objects	3 objects	4 objects
Monkey A	122	85 (70)[1]	155 (32)[2]	45 (29)[3]	20 (13)	10 (6)	10 (6)
Monkey B	83	42 (51)	66 (20)	25 (38)	10 (15)	7 (11)	0 (0)
Both	205	127 (62)	221 (27)	70 (32)	30 (14)	17 (8)	10 (5)

[1]: percentages calculated with respect to total number of neurons in column ‘Total’
[2]: percentages calculated with respect to 488 responses for monkey A, 332 responses for monkey B and 820 responses for both animals.
[3]: percentages calculated with respect to object-value responses in column ‘Responses’.

Table S3. Neuronal responses related to object choice[1], sequential values and other task-relevant variables (related to Figure 4). Results of fitting GLM 5 (Eq. 7) to neuronal activity during sequential object presentation (sliding-window regression). Numbers in parentheses are percentages.

	Self								
	Total	Object 1 first – Object 2 first	Object 3 first – Object 4 first	Object 1 chosen – Object 2 chosen	Object 3 chosen – Object 4 chosen	First object chosen	First object value	Second object value	Chosen object value
Monkey A	122	58 (48)[2]	43 (35)	37 (30)	28 (23)	25 (20)	35 (29)	41 (34)	26 (21)
Monkey B	83	44 (53)	41 (49)	22 (27)	18 (22)	9 (11)	39 (47)	40 (48)	20 (24)
Both	205	102 (50)	84 (41)	59 (29)	46 (22)	34 (17)	74 (36)	81 (40)	46 (22)
	Other								
	Total	Object 1 first – Object 2 first	Object 3 first – Object 4 first	Object 1 chosen – Object 2 chosen	Object 3 chosen – Object 4 chosen	First object chosen	First object value	Second object value	Chosen object value
Monkey A	122	33 (27)	51 (42)	22 (18)	25 (21)	13 (11)	34 (28)	34 (28)	20 (16)
Monkey B	83	34 (40)	47 (56)	24 (29)	15 (18)	13 (16)	40 (48)	41 (49)	12 (14)
Both	205	67 (33)	98 (48)	46 (22)	40 (20)	26 (13)	74 (36)	75 (37)	32 (16)

[1]: The number of choice-coding neurons reported in the main text were obtained from this table from column 'Object 3 chosen – Object 4 chosen'; these objects were the critical objects for observational learning as the partner chose from them at session start while the recorded monkey chose from them after object switch.

[2]: percentages calculated with respect to total number of neurons in column 'Total'

Table S4. Neuronal responses related to object choice, sequential values and other task-relevant variables (related to Figure 6). Results of fitting GLM 6 (Eq. 8) to neuronal activity during sequential object presentation (sliding-window regression). Numbers in parentheses are percentages.

	Total	Object 1 first – Object 2 first	Object 3 first – Object 4 first	Object 1 chosen – Object 2 chosen	Object 3 chosen – Object 4 chosen	First object chosen	First object value	Second object value	Chosen object value	Self – Other
Monkey A	122	60 (49) [1]	65 (53)	30 (25)	33 (27)	25 (20)	44 (36)	44 (36)	16 (13)	99 (81)
Monkey B	83	49 (59)	53 (64)	24 (29)	19 (23)	7 (8)	40 (48)	49 (59)	17 (20)	65 (78)
Both	205	109 (53)	118 (58)	54 (26)	52 (25)	32 (16)	84 (41)	83 (41)	33 (16)	164 (80)

[1]: percentages calculated with respect to total number of neurons in column ‘Total’

Table S5. Neuronal responses related to object choice, sequential values and other task-relevant variables (related to Figure 7). Results of fitting GLM 8 (Eq. 10) to neuronal activity during target presentation (sliding-window regression). Numbers in parentheses are percentages.

	Self									
	Total	Object 1 chosen – Object 2 chosen	Object 3 chosen – Object 4 chosen	First object chosen	First object value	Second object value	Chosen object value	Object 1 left – Object 2 left	Object 3 left – Object 4 left	Left chosen
Monkey A	122	27 (22) [1]	27 (22)	13 (11)	18 (15)	17 (14)	14 (11)	11 (9)	9 (7)	28 (23)
Monkey B	83	12 (14)	14 (17)	5 (6)	17 (21)	18 (22)	13 (16)	21 (25)	11 (13)	14 (17)
Both	205	39 (19)	41 (20)	18 (9)	35 (17)	35 (17)	27 (13)	32 (16)	20 (10)	42 (25)
	Other									
	Total	Object 1 chosen – Object 2 chosen	Object 3 chosen – Object 4 chosen	First object chosen	First object value	Second object value	Chosen object value	Object 1 left – Object 2 left	Object 3 left – Object 4 left	Left chosen
Monkey A	122	6 (5) [1]	14 (11)	4 (3)	16 (13)	16 (13)	9 (7)	7 (6)	13 (11)	3 (2)
Monkey B	83	10 (12)	5 (6)	5 (6)	25 (30)	20 (24)	11 (13)	11 (13)	9 (11)	1 (1)
Both	205	16 (8)	19 (9)	9 (4)	41 (20)	36 (18)	20 (10)	18 (9)	22 (11)	4 (1)

[1]: percentages calculated with respect to total number of neurons in column ‘Total’

Table S6. Neuronal responses related to object value, sequential values and object choice for different amygdala subregions (related to STAR Methods). Numbers in parentheses are percentages.

	Amygdala subregion				
	Total	Lateral	Basolateral	Basomedial	Centromedial
Recorded	205	66	86	23	30
Object value (Eq. 3)	127 (62) [1]	42 (64)	53 (62)	15 (65)	17 (57)
	Self				
First/second object value (Eq. 10)	88 (43)	25 (38)	39 (45)	9 (39)	15 (50)
Object 3 chosen – Object 4 chosen (Eq. 10)	46 (22)	22 (33)	15 (17)	5 (22)	4 (13)
First/second object value AND/OR Object 3 chosen – Object 4 chosen (Eq. 10)	106 (52)	33 (50)	48 (56)	9 (39)	16 (53)
	Other				
First/second object value (Eq. 10)	86 (42)	23 (35)	40 (47)	8 (35)	15 (50)
Object 3 chosen – Object 4 chosen (Eq. 10)	40 (20)	18 (28)	14 (16)	3 (13)	5 (17)
First/second object value AND/OR Object 3 chosen – Object 4 chosen (Eq. 10)	112 (55)	38 (58)	47 (55)	11 (47)	16 (53)

[1]: percentages calculated with respect to number of neurons in row 'Recorded'
